# Phylogenetic and comparative gene expression analysis of barley (*Hordeum vulgare*) WRKY transcription factor family reveals putatively retained functions between monocots and dicots

**DOI:** 10.1186/1471-2164-9-194

**Published:** 2008-04-28

**Authors:** Elke Mangelsen, Joachim Kilian, Kenneth W Berendzen, Üner H Kolukisaoglu, Klaus Harter, Christer Jansson, Dierk Wanke

**Affiliations:** 1 Department of Plant Biology and Forest Genetics, The Swedish University of Agricultural Sciences (SLU), P.O. Box 7080, SE-750 07 Uppsala, Sweden; 2 Center of Plant Molecular Biology (ZMBP), University of Tuebingen, Auf der Morgenstelle 1, D-72076 Tübingen, Germany; 3 CELISCA (Center for Life Science Automation), Friedrich-Barnewitz-Strasse 8, D-18119 Rostock, Germany; 4 Lawrence Berkeley Laboratory, Earth Science Division, 1 Cyclotron Rd., Berkeley, CA 94720, USA

## Abstract

**Background:**

WRKY proteins belong to the WRKY-GCM1 superfamily of zinc finger transcription factors that have been subject to a large plant-specific diversification. For the cereal crop barley (*Hordeum vulgare*), three different WRKY proteins have been characterized so far as regulators in sucrose signaling, pathogen defense, and in response to cold and drought. However, their phylogenetic relationship remained unresolved.

**Results:**

In this study, we used available sequence information to identify a minimum number of 45 barley WRKY transcription factor (HvWRKY) genes. According to their structural features, the HvWRKY factors were classified into the previously defined polyphyletic WRKY subgroups 1 to 3. Furthermore, we could assign putative orthologs of the HvWRKY proteins in Arabidopsis and rice. While in most cases clades of orthologous proteins were formed within each group or subgroup, other clades were composed of paralogous proteins for the grasses and Arabidopsis only, which is indicative of specific gene radiation events. To gain insight into their putative functions, we examined expression profiles of *WRKY *genes from publicly available microarray data resources and found group specific expression patterns. While putative orthologs of the HvWRKY transcription factors have been inferred from phylogenetic sequence analysis, we performed a comparative expression analysis of WRKY genes in Arabidopsis and barley. Indeed, highly correlative expression profiles were found between some of the putative orthologs.

**Conclusion:**

*HvWRKY *genes have not only undergone radiation in monocot or dicot species, but exhibit evolutionary traits specific to grasses. HvWRKY proteins exhibited not only sequence similarities between orthologs with Arabidopsis, but also relatedness in their expression patterns. This correlative expression is indicative for a putative conserved function of related WRKY proteins in monocot and dicot species.

## Background

In plants, WRKY proteins constitute a large family of transcription factors. Since their first identification in sweet potato and wild oats [1,2] the WRKY proteins have been described as transcriptional regulators in multiple plant species and in various processes such as pathogen defense [3-9], trichome [10] and seed development [11], as well as leaf senescence [12,13]. The common feature of WRKY proteins is the existence of either one or two WRKY domains. The WRKY domain is an approximately 60 amino acids long spanning DNA binding domain, which contains a highly conserved amino acid motif, WRKYGQK, at its N-terminus and a metal chelating zinc finger signature at the C-terminus. The crystal structure of one WRKY domain has been resolved recently [14] and provides an insight into how the conserved residues of the WRKY domain facilitate the binding to their cognate DNA-element, the W-box. While most of the functional studies of WRKY proteins indicate a binding to the conserved nucleotide consensus sequence TGAC(C/T) of the W-box, the binding to another cis-element called SURE has also been described [15].

Based on the number of WRKY domains present and on the type of zinc finger WRKY proteins have been classified into groups 1, 2a to 2e and 3 [16]. WRKY transcription factors seem to have their evolutionary origin in ancient eukaryotes with the most basal WRKY genes identified in the unicellular eukaryote *Giardia lamblia *and the slime mold *Dictyostelium discoideum *[17,18]. While the WRKY-GCM1 superfamily has been found in all organism kingdoms, the conserved WRKY-type signatures of these genes have not yet been detected in the lineage of true fungi and animals [19]. In contrast, phylogenetic analysis revealed that the WRKY transcription factor family has expanded to great extent in higher plants [20]. For example, the WRKY gene family consists of 72 members in Arabidopsis [16] and at least 81 members in rice [6,18,21,22].

The cereal crop barley is of high importance for food, feed and brewing. After maize, rice and wheat, barley ranked as number four in cereal production in 2005 [23,24]. Due to its wide ecological potential and adaptability, it is cultivated worldwide and under diverse environmental conditions. For example, barley is grown on soils and in altitudes that are unsuitable for wheat and oats [23,24]. The genome of barley is estimated to be of approximately 5000 Mb in size and has not been fully sequenced yet. However, the GenBank EST dataset for barley contains almost 440,000 ESTs and provides a valuable resource for bioinformatics analyses [25]. In addition, the Affymetrix 22 K Barley1 GeneChip microarray, representing probe sets for roughly 22,000 genes [26] was released in 2004. Nowadays, a number of barley microarray experiments are accessible via different public sources such as BarleyBase [27,28] or GEO [29,30] and allow for *in silico *expression analyses of barley genes.

Several studies have shown an interest in barley WRKY proteins (HvWRKY) and their function. HvWRKY1, also named HvWRKY38, has been reported to be involved in several regulatory processes, such as the response to cold and drought [31] and the repression of the alpha-amylase gene *Amy32b *during seed germination [32]. Recently, the same WRKY protein and its paralog HvWRKY2 have been described as repressors of the PAMP-triggered basal defense upon infection with *Blumeria graminis *[8]. Apart from HvWRKY proteins functioning in response to biotic and abiotic stresses, the group 1 WRKY protein SUSIBA2 has been shown to mediate sucrose signaling in barley cells during regulation of starch synthesis [15,33].

In this study we identified 45 members of the *WRKY *gene family in barley based on publicly available sequence information. We describe their phylogenetic relationship and try to assign putative orthologs from barley, Arabidopsis and rice. Expression analysis using microarray data suggests the involvement of some of the HvWRKY proteins in response to powdery mildew and in plant development. Moreover, correlated expression of orthologous barley and Arabidopsis WRKY genes imply that their function is retained in monocot and dicot species.

## Results

### Identification of 45 WRKY protein coding sequences in barley

To estimate the minimum number of *WRKY *genes in barley, publicly available sequence data was searched with the BLAST program. The protein sequence of WRKY domains from representative members for all subgroups from Arabidopsis (AtWRKY1, -18, -6, -8, -7, -14 and -30) were used as input. The primary search resulted in 142 non-redundant hits, of which 20 were removed as they did not contain the conserved WRKY domain signature. The remaining 122 sequences were screened for partial overlaps by pairwise comparison; this analysis identified 45 WRKY open reading frames (ORFs) unique for barley. A keyword search on NCBI returned eight previously annotated HvWRKY protein sequences. As indicated by corresponding EST sequences, all eight ORFs were expressed. In order to create a uniform nomenclature with consecutive numbering, we renamed sequences annotated as putative WRKY2, 3, 4, 5 and 6 to HvWRKY2, 3, 4, 5 and 6, respectively. Sequences of HvWRKY3 and 4 could be extended based on available EST information. The sequence of putative WRKY1 protein, CAD60651, is identical with the sequence AAS48544 which has been published as HvWRKY-38 [31]. We designated both sequences as HvWRKY1 and did not annotate any sequence as HvWRKY38 to prevent confusion. SUSIBA2 received the formal name HvWRKY46. The remaining sequences were annotated accordingly as HvWRKY7 to HvWRKY37 and HvWRKY39 to HvWRKY45 (Table 1). Seven of the HvWRKY sequences included start and stop codons and, as judged by protein sequence alignments with WRKY proteins from rice (data not shown), constitute complete coding sequences. The remaining 39 HvWRKY sequences represented partial coding sequences. To confirm that we were identifying clade members correctly, we extended sequence information by PCR HvWRKY6 and HvWRKY9, a putative group 1 WRKY and group 2d WRKY, respectively. This approach revealed a second WRKY domain in the sequence of HvWRKY6, confirming that HvWRKY6 is indeed a class 1 member. In case of HvWRKY9, a characteristic domain with the highly conserved amino acid sequence HARF [34] could be detected at the beginning of the coding sequence confirming that it too, was correctly assigned by our analysis.

**Table 1:** Identified WRKY proteins in barley and their putative orthologs in Arabidopsis and rice based on phylogenetic studies of their respective WRKY domain sequences.

**HvWRKY**	**Accession number**	**WRKY group**	**AtWRKY ortholog**	**OsWRKY ortholog**
HvWRKY1/38	AJ536667	IIa	18,40,60	28,71
HvWRKY2	AJ853838	IIa	18,40,60	28,71
HvWRKY3	EF488104	IIa	18,40,60	76
HvWRKY4	EF488105	III	54,70	47
HvWRKY5	AJ853841	IIc	50	77
HvWRKY6	EF488106	I	1	82
HvWRKY7	DQ840406	IId	11,17	68
HvWRKY8	DQ840407	IId	39,74	*
HvWRKY9	DQ840408	IId	7	*
HvWRKY10	DQ840409	IId	*	51
HvWRKY11	DQ840410	IId	*	6
HvWRKY12	DQ840411	IIc	75	72
HvWRKY13	DQ840412	IIc	56	23
HvWRKY14	DQ840413	IIc	13	79
HvWRKY15	DQ840414	IIc	57	3,29
HvWRKY16	DQ840415	IIc	50	67
HvWRKY17	DQ840416	IIc	50	10
HvWRKY18	DQ840417	IIc	50	26,59
HvWRKY19	DQ840418	IIc	50	7
HvWRKY20	DQ840419	IIc	50	7
HvWRKY21	DQ863105	III	30,41,53	15
HvWRKY22	DQ863106	III	30,41,53	74
HvWRKY23	DQ863131	IIa	18,40,60	28,71
HvWRKY24	DQ863108	I	30,41,53	63
HvWRKY25	DQ863109	III	30,41,53	81
HvWRKY26	DQ863110	III	30,41,53	15
HvWRKY27	DQ863111	III	30,41,53	19
HvWRKY28	DQ863112	III	30,41,53	74
HvWRKY29	DQ863113	III	30,41,53	15
HvWRKY30	DQ863114	III	30,41,53	69
HvWRKY31	DQ863115	III	54,70	48
HvWRKY32	DQ863116	III	54,70	45
HvWRKY33	DQ863117	III	*	46
HvWRKY34	DQ863118	III	*	46
HvWRKY35	DQ863119	I	19	*
HvWRKY36	DQ863130	IIc	49	17
HvWRKY37	DQ863121	IIb	72	73
HvWRKY39	DQ863122	IIe	65	13
HvWRKY40	DQ863123	I	44	38
HvWRKY41	DQ863124	I	*	*
HvWRKY42	DQ863125	I	2	80
HvWRKY43	DQ863126	I	33	24
HvWRKY44	DQ863129	IIe	22	39
HvWRKY45	DQ863128	IIe	69	14
HvWRKY46 (Susiba2)	AY323206	I	20	78

* ortholog could not be assigned with significance

**Figure 1:**
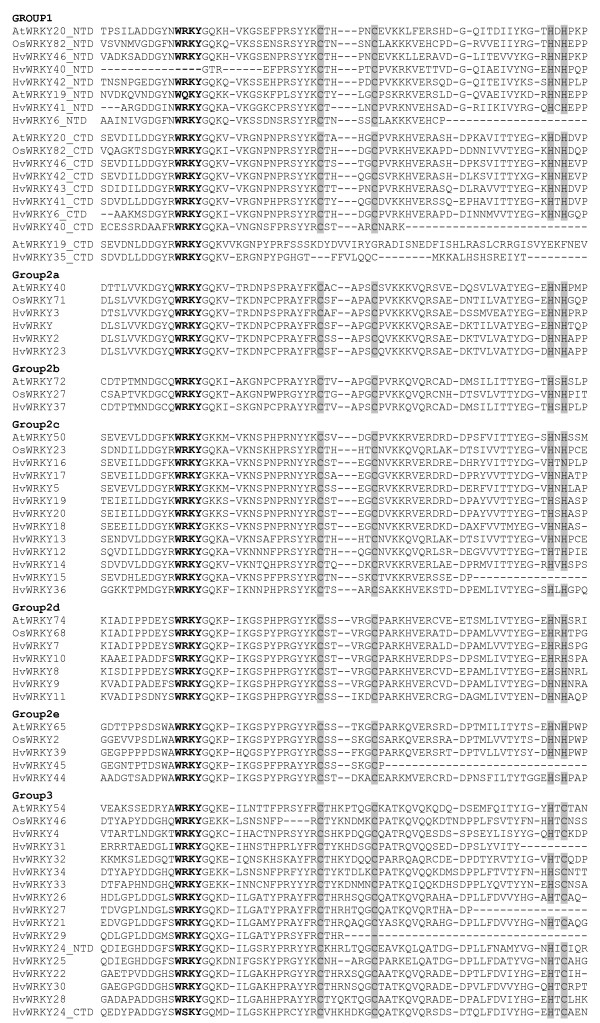
**Multiple alignment of the identified barley (HvWRKY) and selected Arabidopsis (AtWRKY) and rice (OsWRKY) WRKY domain sequences**. N-terminal and C-terminal WRKY domains of group 1 WRKY proteins are indicated as _NTD and _CTD, respectively. The conserved WRKY amino acid signature is highlighted in bold letters, the amino acids forming the zinc-finger motif are displayed in grey, gaps are marked with dashes.

While Eulgem et al. [16] defined WRKY proteins by the conserved heptapeptide WRKYGQK, slight variations of this sequence have been described for a number of rice WRKY proteins [20,21]. Similarly, a number of HvWRKY sequences have amino acid sequence substitutions in the conserved WRKY signature. In HvWRKY18, 19 and 20 the WRKY domain displays the sequence WRKYGKK, in HvWRKY33 and 34 WRKYGEK, in HvWRKY36 WRKYGQN and in the C-terminal domain of HvWRKY24 WSKYGQM (Figure 1). Moreover, a unique zinc-finger motif (C-X_4–5_-C-X_22–23_-H-X-H) was described as a second characteristic feature of WRKY proteins[16]. In group 3 WRKY proteins, this motif is slightly altered to C-X_7_-C-X_23_-H-X-C. In barley, three of the so far characterized group 3 WRKY proteins, namely HvWRKY33 and 34, showed an extension of the zinc finger motif (C-X_7_-C-X_24_-H-X-C) compared to the patterns described by Eulgem et al. [16] (Figure 1). Again, similar modifications of the zinc-finger patterns were detected for rice WRKY proteins and have been discussed to be a monocot-specific feature [18,20,21].

### Phylogenetic analysis of barley WRKY proteins

While the phylogenetic analysis showed that WRKY proteins are of monophyletic nature [19], the assignment to defined groups is polyphyletic and either based on the number of WRKY domains or on the type of zinc finger motif. To study the phylogenetic relationship of the 45 HvWRKY proteins we performed a multiple sequence alignment of the about 60 amino acids spanning the WRKY domain of all HvWRKYs. To obtain a better separation of the different groups and subgroups, WRKY domains from members of different Arabidopsis and rice WRKY proteins were included in the analysis. For each of the groups 1, 2a to 2e and 3 one representative was chosen randomly. These were: AtWRKY20, -40, -72, -50, -74, -65, -54 for Arabidopsis and OsWRKY82, -71, -27, -25, -68, -2, -46 for rice (classified according to Xie et al. [21]). Both C-terminal and N-terminal WRKY domains of group 1 WRKY proteins have been treated as independent sequences, so that a total number of 51 barley, 8 Arabidopsis and 8 rice WRKY domains were analyzed.

**Figure 2:**
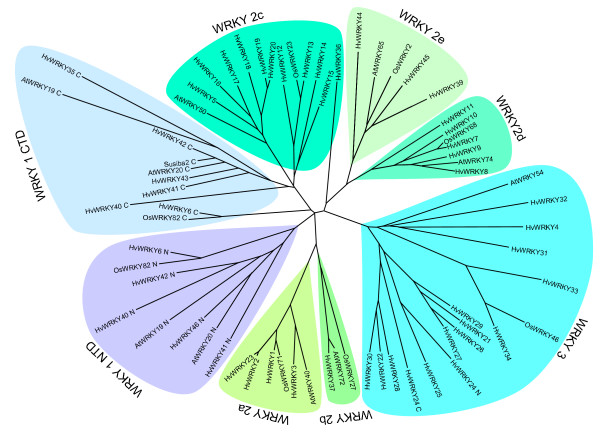
**Unrooted phylogenetic tree of identified barley WRKY proteins.** The tree was calculated on the basis of WRKY domain sequences of the barley (HvWRKY) and selected Arabidopsis (AtWRKY) and rice (OsWRKY) protein sequences as representatives for the different groups. WRKY groups and subgoups 1 to 3 are highlighted in different colors. Color coded grouping of HvWRKY36, HvWRKY6 and OsWRKY82 is based on analysis with sequences extending over the WRKY domain consensus.

As shown in Figure 2 for all subgroups of WRKY proteins, we could identify at least one representative in the barley genome. For example, five HvWRKY sequences code for proteins with two WRKY domains and clearly group with the group 1 members AtWRKY2 and OsWRKY82. The single WRKY domain identified for HvWRKY43 clusters within the C-terminal group 1 domains, indicating that a second N-terminal domain remains likely to be identified. Interestingly, the 24 identified group 2 WRKY members of barley are distributed very unevenly among the five subgroups. Whereas 11 HvWRKYs form a distinct subclade with the characteristic members of subgroup 2c, only a single barley WRKY, HvWRKY37, belongs to group 2b. Group 3 is represented by 13 single-WRKY domain barley proteins yet, both WRKY domains of HvWRKY24 cluster with these proteins. Again, this phenomenon has been observed for at least four rice WRKY proteins: OsWRKY41, -61, -63 and -81 [21].

The total number of barley WRKY genes will remain unknown until the sequencing of the barley genome is completed. However, we constructed an alignment and a phylogenetic tree of 72 Arabidopsis, 45 barley and 81 rice WRKY proteins to estimate this number (Additional Files 1 and 2). As described earlier by Zhang and Wang [20], group 2 and 3 domains were closer related to the C-terminal domains of group 1 whereas the N-terminal WRKY domains of group 1 members form a separate monophyletic cluster. On the other hand, separate subclades of the WRKY domains of subgroup 2c underline the paraphyletic nature of these subgroups (Additional File 2).

The grouping of HvWRKY36, HvWRKY6 and OsWRKY82 based solely on the phylogenetic analysis of their WRKY domain sequences can not be performed unambiguously. However, the presence of two WRKY domains in HvWRKY6 and OsWRKY82 is indicative for group 1 members. Both C-terminal and N-terminal WRKY domains of HvWRKY6 and OsWRKY82 support basal monophyletic subclades of group 1 WRKY proteins (Figure 1 and Additional File 2), which might be specific for grasses [21].

The number of WRKY proteins within each species assigned to the different paraphyletic subgroups 1 to 3 was calculated and is summarized in Table 2. A simple comparison of the total number of Arabidopsis and rice WRKY genes with the number of HvWRKYs indicates that approximately 50% of the barley WRKY genes have been identified. Also, the distribution of WRKY proteins showed that HvWRKYs are underestimated in several subgroups. Nevertheless, similar numbers of barley and rice WRKY proteins in subgroups 2a and 2d may indicate that all HvWRKY proteins of this subgroup have been identified.

**Table 2:** Number of WRKY proteins of Arabidopsis (AtWRKY), rice (OsWRKY) and barley (HvWRKY) in the WRKY subgroups 1 to 3*.

**WRKY group**	**AtWRKY**	OsWRKY**	**HvWRKY**
1	15	16	8
2a	3	4	4
2b	8	7	1
2c	17	20	11
2d	7	6	5
2e	8	8	3
3	14	20	13

**Total**	**72**	**81**	**45**

* according to Eulgem et al. [16]

**according to Xie et al. [21]

### Monocot-specific radiation and identification of orthologous *WRKY *genes

Based on the WRKY domains of all proteins from barley, Arabidopsis and rice, we calculated a comprehensive phylogenetic tree to gain a closer insight into the evolutionary relationships between WRKY proteins (Additional File 2) and assign putative WRKY orthologs between these sequences. Additionally, nine WRKY domains of the moss *Physcomitrella patens*, two each from *Giardia lamblia *and *Dictyostelium discoideum*, as well as additional zinc finger domains of other WRKY-GCM1-like proteins were included as outgroups.

Clades exist for almost all WRKY subgroups which consist solely of rice WRKY domains or a combination of rice and Arabidopsis WRKY domains; these clades might be comprised of orthologs of yet unidentified barley WRKY proteins. In contrast, rice and barley WRKY domains form a number of monocot-specific clades inside group 2c and group 3. These clades reveal clusters of orthologous WRKY proteins for rice and barley. For example in subgroup 2c, AtWRKY50 forms a basal accession to five barley and six rice WRKY domains. Similarly, in a subgroup of group 3, ten barley and nine rice WRKY proteins constitute putative monocotyledonous orthologs that diverge from a group of three paralogous Arabidopsis WRKY proteins (Additional File 2). Thus, the barley and rice WRKY proteins that form paralogous or orthologous groups would indicate monocot-specific diversification of WRKY genes. This assumption was further supported by BLAST searches in dbEST [25], which did not return orthologous WRKY domains of any other dicotyledonous species for the monocot-specific subclades (data not shown). Thus, our results support the idea of radiation events in groups 2c and 3 WRKY proteins that are specific for monocot species. This diversification has most likely occurred after the ancestral divergence of dicotyledonous and monocotyledonous plants.

The assignment of orthologs is crucial for transferring the knowledge of WRKY protein functions from the model plants rice and Arabidopsis to barley. Therefore, based on the presented phylogenetic tree (Additional File 2), we assigned orthologs for most of the WRKY genes described (Table 1). In a few cases orthologs could not be defined unambiguously. However, as gene orthology implies similar gene function, the assignment presented provided a solid base for functional analyses.

### Powdery mildew infection affects expression of *HvWRKY *genes

**Figure 3:**
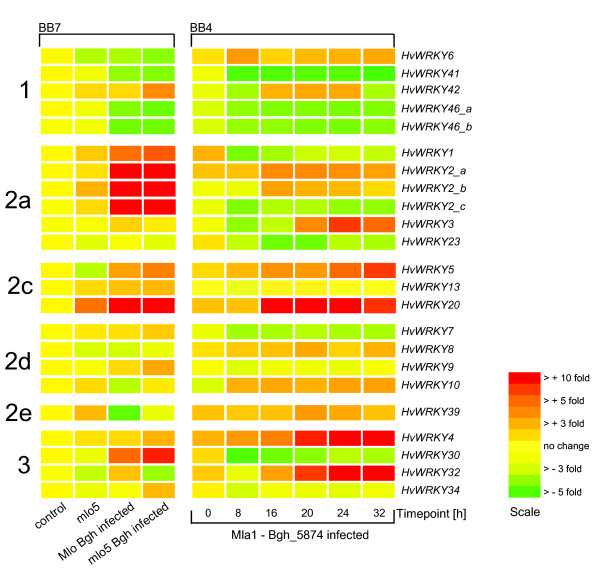
***HvWRKY *****gene expression upon infection with***** Blumeria graminis *****(Bgh).** Normalized signal intensities of 23 probesets representing *HvWRKY *genes are displayed for microarray experiments BB4 and BB7 (according to BarleyBase [27, 28]). Experimental samples and timepoints are indicated on the x-axis. Mlo, mlo5 and Mla1 represent different barley genotypes, Bgh_5874 represents a particular strain of powdery mildew (see BarleyBase [27, 28] for experimental details). Fold changes compared to the control (BB7) or timepoint zero (BB4) are color coded as indicated. *HvWRKY *gene probesets are arranged according to WRKY groups 1 to 3. Several probesets representing the same gene are named _a to _c.

The analysis of temporal and spatial expression patterns is an initial step towards the functional characterization of genes. The expression of numerous WRKY genes from various plant species is affected by biotic and abiotic stresses ([6] and references therein) and implies the regulatory role of WRKY proteins in stress responses. In order to investigate if this is also true for *HvWRKY *genes, we mined publicly available microarray data of barley leaves infected with powdery mildew (*Blumeria graminis*). A total of 23 probesets on the Barley1 GeneChip could be assigned to 20 different *HvWRKY *genes (Table 3). Datasets of two experiments named BB4 and BB7 were retrieved from BarleyBase [27,28,35] and analyzed. Figure 3 shows the expression profiles of 20 *HvWRKY *genes are displayed for two genotypes (Mlo and mlo5) under infection and non-infection conditions (BB7) as well as during a time course experiment using the host genotype Mla1 and the Bgh isolate 5874 (BB4, see publication for details). Even though the setup of the BB7 and BB4 experiments differ, some general trends can be seen in both experiments. Roughly 50% of all *HvWRKY *genes show altered expression levels upon infection with *Blumeria graminis*. HvWRKY1 and HvWRKY2 have been previously shown to be involved in the regulation of defense mechanisms [8]. *HvWRKY1 *expression appears to be drastically increased under one experimental condition (BB7) and lower in the second (BB4), while *HvWRKY2 *shows increased expression levels in both experiments. In addition to these two WRKY genes, a number of other *HvWRKY *genes mediating pathogen responses could be assigned based on their expression profiles. For example, the gene for HvWRKY3, which is closely related to the well-studied pathogen response regulators AtWRKY18, -40 and -60, shows an increase in expression after 20 h of pathogen treatment. Also, putative pathogen response regulators could be found in subgroups 2c and 3. Interestingly, these are WRKY groups that have undergone monocot-specific radiation events, which might thus have been related to an increased biotic stress during evolution. In addition to upregulated genes, there are *HvWRKY *genes with decreased transcript levels, such as most of the *HvWRKY *genes in group 1. However, expression of several *HvWRKY *genes was not affected upon powdery mildew infection and, therefore, might function in regulatory processes other than pathogen response such as abiotic stress or development.

**Table 3:** Probesets of *HvWRKY *genes on the Barley1 GeneChip.

***HvWRKY***	**WRKY group**	**Barley1 Chip probeset**
*HvWRKY6*	I	CONTIG14308_AT
*HvWRKY41*	I	CONTIG12033_AT
*HvWRKY42*	I	CONTIG15657_AT
*HvWRKY46 (Susiba2)*	I	CONTIG7243_AT; RBAAL15J13_S_AT
*HvWRKY1 (HvWRKY-38)*	IIa	CONTIG4386_AT
*HvWRKY2*	IIa	CONTIG4387_AT; HB25K10R_S_AT; HB25K10R_AT
*HvWRKY3*	IIa	EBRO02_SQ004_H10_AT
*HvWRKY23*	IIa	CONTIG21110_AT
*HvWRKY5*	IIc	CONTIG18462
*HvWRKY13*	IIc	CONTIG13268_AT
*HvWRKY20*	IIc	CONTIG10168_AT
*HvWRKY7*	IId	CONTIG7798_AT
*HvWRKY8*	IId	CONTIG23011_AT
*HvWRKY9*	IId	CONTIG22226_AT
*HvWRKY10*	IId	CONTIG16040_AT
*HvWRKY39*	IIe	CONTIG13375_AT
*HvWRKY4*	III	CONTIG20450_AT
*HvWRKY30*	III	CONTIG12005_AT
*HvWRKY32*	III	S0001000055P18F1_S_AT
*HvWRKY34*	III	CONTIG10471_AT

### Orthologous barley and Arabidopsis *WRKY *genes exhibit correlative expression signatures during plant development

A number of WRKY proteins have been reported to be involved in plant developmental processes [10-12]. To investigate if *HvWRKY *transcript levels vary during plant development, we used public microarray data of different developmental stages from barley [36] and investigated whether phylogenetically related *WRKY *genes of barley and Arabidopsis exhibited similarities in their expression patterns. As not all *WRKY *genes of both barley and Arabidopsis are represented on their respective microarrays, we were restricted to the expression patterns of 16 *HvWRKY *genes and their putative Arabidopsis orthologs. We extracted expression data from the total microarray dataset for ten organs in barley at different developmental stages:coleoptile, mesocotyl, radicule, leaf, root, inflorescence, anthers, and caryopsis 5, 10 and 16 days post anthesis (d.p.a.) [36].

**Figure 4:**
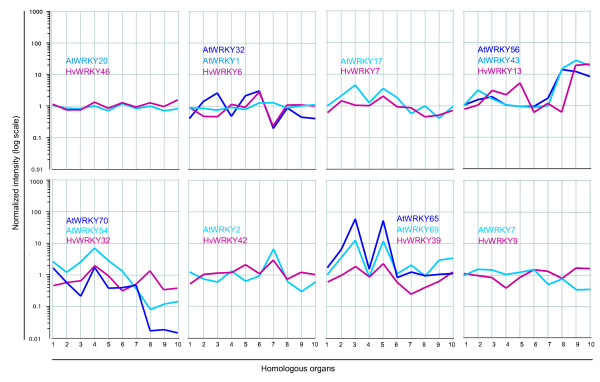
**Selected expression profiles of barley *WRKY *genes (*HvWRKY*) and their designate orthologs in Arabidopsis (*AtWRKY*)**. The signal intensities of the developmental baseline experiments by Druka *et al*. [36] and Schmid *et al*. [37] are normalized to the mean expression values and are plotted in log-scale for different homologous plant organs (1–10). The respective organs of barley and Arabidopsis are: 1 (coleoptile/cotyledon), 2 (mesocotyl/hypocotyl), 3 (radicule/seedling roots), 4 (leaf/leaf), 5 (root/root), 6 (inflorescence/inflorescence), 7 (anthers/stamen), 8 (caryopsis 5dpa/seed stage 6), 9 (caryopsis 10dpa/seed stage 8) and 10 (caryopsis 15dpa/seed stage 10).

The expression profiles reveal spatial and temporal variations in expression of *HvWRKY *genes in different barley organs (Additional File 3). Some genes, like *HvWRKY2*, -*4 *and -*46*, have similar expression levels in all investigated tissues. In contrast, genes such as *HvWRKY5*, -*6*, -*9*, -*39 *and -*41 *reveal more than 3-fold difference in expression levels in different organs. Furthermore, the transcript levels of barley *WRKY *genes in the same organ show temporal variations. For example, *HvWRKY32 *is expressed in young caryopses at 5 d.p.a. but is clearly reduced in later stages of caryopsis development (Figure 4). Similarly, the expression levels of *HvWRKY6 *are low in radicules but significantly higher in older roots. In addition, paralogous barley *WRKY *genes such as *HvWRKY5 *and -*20 *reveal similar expression patterns, implying redundant function.

Next, we performed a comparative expression analysis for both barley and Arabidopsis *WRKY *genes on the extracted subsets of the microarray expression datasets of homologous organs by Druka et al. [36] and Schmid et al. [37].

The Pearson correlation of the normalized signal intensities was calculated for the potentially orthologous *WRKY *genes. The resulting correlation coefficients ranged from -0.49 to +0.91 (Table 4). The average correlation of all the putative orthologous *WRKY *genes was +0.24 and differed significantly (*p*-value 0.009) from the average expression correlation of a control dataset composed of 101 randomly chosen gene pairs (+0.01; Additional File 3). Selection of only the best correlated pairs of *HvWRKY *and *AtWRKY *orthologs even increased the average correlation coefficient to +0.37. This high correlation can be easily seen by the overall expression pattern of the compared *WRKY *genes (Figure 4). For example, the expression levels of the orthologous pair in group 1, *AtWRKY20 *and *HvWRKY46*, hardly differ in any of the ten organs and are clearly correlated. In contrast, the expression profiles of HvWRKY9 and AtWRKY7 are observably anti-correlated. Genes for the group 2e WRKY protein HvWRKY39 and their closely related Arabidopsis orthologs AtWRKY65 and -69 show a consistent increase in mRNA level in radicules and roots with low expression levels in leaves. According to our phylogenetic analysis, HvWRKY6 has two potential orthologs in Arabidopsis, namely AtWRKY1 and AtWRKY32. The *HvWRKY6 *expression profile resembles that of *AtWRKY1 *(+0.41) in vegetative tissues but is more similar to the profile of *AtWRKY32 *(+0.45) in reproductive organs such as the inflorescence and the anthers. This might indicate that the Arabidopsis *WRKY *genes have undergone functional changes after a duplication event.

**Table 4:** Pearson correlation coefficients of the expression profiles of *HvWRKY *genes and their designate *AtWRKY *orthologs.

***HvWRKY***	***AtWRKY***	**Correlation coefficient**
*HvWRKY1*	*AtWRKY18*	0.06
	*AtWRKY40*	-0.11
	*AtWRKY60*	-0.18
*HvWRKY2*	*AtWRKY18*	-0.05
	*AtWRKY40*	-0.10
	*AtWRKY60*	-0.31
*HvWRKY3*	*AtWRKY18*	0.21
	*AtWRKY40*	-0.24
	*AtWRKY60*	-0.11
*HvWRKY4*	*AtWRKY54*	0.52
	*AtWRKY70*	0.23
*HvWRKY6*	*AtWRKY1*	0.41
	*AtWRKY32*	0.45
*HvWRKY7*	*AtWRKY11*	0.38
	*AtWRKY17*	0.66
*HvWRKY8*	*AtWRKY39*	0.20
	*AtWRKY74*	0.30
*HvWRKY9*	*AtWRKY7*	-0.49
	*AtWRKY15*	0.12
*HvWRKY13*	*AtWRKY43*	0.82
	*AtWRKY56*	0.54
*HvWRKY23*	*AtWRKY30*	0.33
	*AtWRKY53*	0.39
*HvWRKY28*	*AtWRKY30*	-0.12
	*AtWRKY53*	-0.07
*HvWRKY32*	*AtWRKY54*	0.70
	*AtWRKY70*	0.42
*HvWRKY34*	*AtWRKY55*	-0.37
*HvWRKY39*	*AtWRKY65*	0.88
	*AtWRKY69*	0.91
*HvWRKY42*	*AtWRKY2*	0.78
*HvWRKY46*	*AtWRKY20*	0.50

Average correlation all WRKY gene pairs		0.24
Average correlation best correlated WRKY gene pairs*		0.37
Average correlation random genes**		0.01

* For each *HvWRKY *gene, the *AtWRKY *gene with the highest correlation value was chosen to calculate the average correlation of best pairs.

** Correlation of 101 random genes of barley and Arabidopsis. Probesets on the Barley1 GeneChip and ATH1 GeneChip were randomly selected and the average of the Pearson correlation of their expression profiles was calculated.

**Figure 5:**
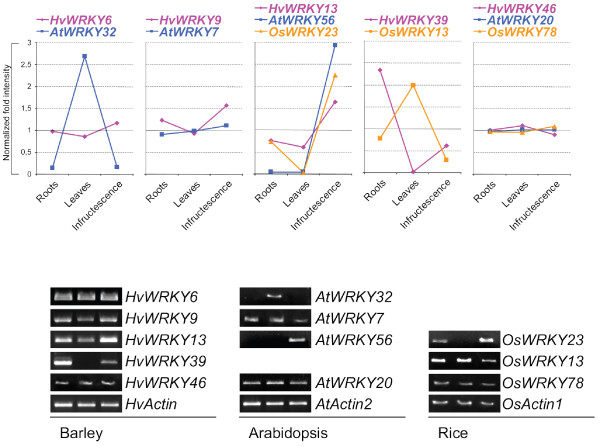
**Expression of selected barley *WRKY *genes (*HvWRKY*) and their designate orthologs in Arabidopsis (*AtWRKY*) or rice (*OsWRKY*)**. The normalized fold difference (upper diagram) of the signal intensities of each amplicon has been calculated from the RT-PCR experiments (lower diagram) to estimate the expression trajectories of *WRKY *genes relative to its *Actin *controls over three different organs in three plant species. RT-PCR analysis (lower panels) with mRNA of barley, Arabidopsis and rice isolated from homologous organs (roots, left; leaves, middle; infructescence, right).

Next, we performed RT-PCR experiments on cDNA from three different organs (roots, leaves and infructescences) and to extend our studies we also included samples from rice, *Oryza sativa japonica*, for the analysis. The signal intensities of the PCR products were quantified and normalized to the *Actin *controls to calculate normalized fold intensities for each organ and organism (Additional File 4). First of all, the expression trajectories of all tested barley and Arabidopsis genes generally reproduced those observed in the microarrays experiments (Figure 5). Transcripts of the three orthologs *HvWRKY46*, *OsWRKY78 *and *AtWRKY20 *were the most abundant and expressed to the same amount in all organs. Although *HvWRKY39 *and *OsWRKY13 *constitute close relatives, their normalized intensities differ especially in leaves where no *HvWRKY39 *transcript could be detected (Figure 5).

Intriguingly, the expression of the closely related group 2c *WRKYs HvWRKY13*, *OsWRKY23, AtWRKY43 *and *AtWRKY56 *was highly correlated and strongly increased in seed stages compared to leaves (Figure 4 and 5). From this we can conclude that these four WRKY genes are of high general importance in all of the three plants for seed development as the comparative organs, siliques and caryopses or infructescences, differ greatly in their structure and composition.

## Discussion

In this study we present the annotation of 45 members of the *WRKY *gene family in barley and classify them according to the WRKY groups 1 to 3 (Figures 1 and 2, Table 1). Comparison to the total number of *WRKYs *in Arabidopsis and rice (Table 2) allowed us to identify about 50% of all barley *WRKY *genes. This is in accordance with a study by Ryu and colleagues [6] who found EST sequences for 44% of all *OsWRKY *genes.

In order to transfer the knowledge of WRKY function in Arabidopsis and rice to barley, we created a phylogenetic tree including the WRKY protein sequences of all three species. For example, AtWRKY7 (group 2d) has been shown to bind to calmodulin in a Ca^2+^- dependent manner by a calmodulin-binding domain (CaMBD), which has been found in all other group 2d WRKY proteins of Arabidopsis [34]. Following our phyogenetic analysis, HvWRKY9 is the ortholog of AtWRKY7 (Additional File 2) and might therefore have similar calmodulin-binding capacity. Even though the current sequence of HvWRKY9 does not span the CaMBD, we could identify such a CaMBD for HvWRKY7 and HvWRKY10 which are members of the same subclade. Also, a sequence alignment of all group 2d OsWRKY proteins revealed the CaMBD in four of six OsWRKY proteins (data not shown), arguing for a conserved function of this domain in combination with the WRKY binding domain in angiosperms. Interestingly, although most identified WRKY clades have at least one member involved in pathogen stress, none of the group 2d *HvWRKY *genes displayed significant changes in expression levels upon powdery mildew infection (Figure 3). This underlines the uniformity of group 2d HvWRKY proteins, and also leads to the speculation whether these HvWRKY proteins are impaired in the regulation of pathogen response in a calmodulin-binding dependent manner.

For rice and Arabidopsis members of WRKY group 2a an involvement in the response to fungal pathogens has been demonstrated [6,38]. Recently, HvWRKY1 and HvWRKY2 have been shown to play an important role in the regulation of the response to the fungal pathogen *Blumeria graminis *[8]. Our alignment tree indicates the existence of two further group 2a WRKY proteins in barley, HvWRKY3 and HvWRKY23, which therefore are also potential regulators of pathogen response. In fact, the expression levels of all group 2a *HvWRKY *genes alter upon powdery mildew infection (Figure 3). Interestingly, three out of six rice members of the monocot-specific subclade of group 2c having AtWRKY50 as a basal member show increased expression levels upon infection with the fungus *Magnaporthe grisea *[6]. The same is true for at least two other 2c barley members, namely *HvWRKY5 *and HvWRKY20, which show changes in expression levels in *Blumeria graminis *challenged leaves (Figure 3). This could indicate that the diversification of this particular subclade of WRKY transcription factors might have occurred in response to pathogen stress. As all identified pathogen-responsive OsWRKYs (OsWRKY7, -10, -67) so far have corresponding orthologs in barley, these proteins are also potential regulators of pathogen response.

Xie and co-workers [39] showed synergistic interactions of OsWRKY71 (group 2a) and OsWRKY51 (group 2d) in regulation of *Amy32b *gene expression in rice aleurone cells and parts of their work was used for a comparative analysis in barley, where HvWRKY1 (HvWRKY38) was shown to be involved in regulating *Amy32b *expression [32]. In fact, based on our alignment, OsWRKY71 and HvWRKY1 were shown to be putative orthologs. However, an interaction partner of HvWRKY1 with functional similarities to OsWRKY51 has not been described so far [32], although one may postulate that the ortholog to OsWRKY51, HvWRKY10, constitutes a good candidate as an interaction partner to HvWRKY1. In summary, the sequence comparison of WRKY proteins of different species aids in assigning functions to different WRKY proteins and allows for target-oriented hypothesis testing.

Microarray-based studies have become a highly valuable source for functional genomics [40] and huge publicly available datasets allow scientists to monitor the expression of their genes of interest. In this study, we performed a cross-species expression analysis using the microarray data of developmental studies in barley and Arabidopsis [36,37]. We considered similarities in expression profiles as an indicator for conserved functional similarities of orthologous genes. Our study was limited by the fact that only 20 of the *HvWRKY *genes and 61 of the *AtWRKY *genes are represented by probesets on the respective microarrays. Also, there was an obvious risk of not comparing true orthologs as only about 50% of all *HvWRKY *genes could be identified (Table 2). Although cultivation of barley and Arabidopsis plants differed, the homologous organs we considered are known to harbor plant lineage-specific functions (see [36,37]); we observed an average positive Pearson correlation of +0.24 in the gene expression of putative *WRKY *orthologs between these two species (Table 4, Figure 4). This is consistent with the results of genome-wide expression comparisons of five developmental stages of Arabidopsis and rice, which returned correlation coefficients in the same range [41]. Similarly, a comparative study of the expression of members of the basic helix-loop-helix transcription factors in Arabidopsis and rice indicates conserved expression patterns of orthologs in four corresponding plant organs [42], further indicating that protein function is likely conserved between homologous organs. When only considering the best pairing WRKY orthologs, the average Pearson correlation for gene expression increased to +0.37. This is mostly due to a reduction in the number of putatively orthologous gene-pairs exhibiting no correlation (Additional File 5). Several reasons can be suggested which might explain why some of these putative orthologs of barley and Arabidopsis show random or negatively correlated expression profiles. First, the function of the orthologous *WRKY *genes might have changed during evolution, mirrored by changes in expression patterns. Moreover, the expression of these *WRKY *genes might not be related to any developmental processes and instead are only activated upon abiotic and biotic stresses. Since the limitation of identifying orthologs between barley and Arabidopsis was based on the available barley EST data, it is possible that a better HvWRKY ortholog may exist for each AtWRKY in our current assignment. It is possible, therefore, that poor correlations observed in this study could be improved once more EST or genomic sequence is available. Nevertheless, the current phylogenetic assignments cover and establish a solid orthology for future studies.

In this study we have shown the potential value of comparative expression analysis between sequenced model species and non-sequenced species of high economic impact. Nowadays, microarrays for several non-sequenced crop species are commercially available. Thus, the reported combination of phylogenetic and expressional analysis may offer a valuable tool for future studies and may contribute to the understanding of gene functions in these crops.

## Conclusion

The basis for deriving orthology between related proteins of different plant species is their degree in similarity. Here, we could provide that this orthology is not restricted to sequence similarities alone but reaches out to orthology in expression patterns. We speculate that this observation is not restricted to the WRKY proteins based on observations made in studies of other gene families. Nonetheless, the combination of phylogenetic characterization of *HvWRKY *genes and the subsequent comparative expression analysis of orthologous genes in Arabidopsis provides a solid basis for further comparative functional genomics studies of this gene family in the cereal crop barley.

## Methods

### Database search and sequence annotation

To find ESTs coding for WRKY proteins in barley (*Hordeum vulgare*) we performed a tBLASTn search [43,44] on the NCBI dbEST dataset for barley [25]. The WRKY domain sequences of seven Arabidopsis (*Arabidopsis thaliana*) WRKY family members (AtWRKY1,18, 6, 8, 7 14 and 30) each representing one of the WRKY subgroups described by Eulgem et al. [16] were used as query sequences. Blast default settings were used except the low complexity filter was deselected. The 142 best hits were processed further. To confirm that the obtained cDNA sequences encoded WRKY proteins, the nucleotide sequences were translated into amino acid sequences, which were then examined for the existence of the heptapeptide WRKYGEK and its variations [20] 20 sequences were removed as they did not contain any WRKY sequence. The remaining ESTs were aligned using ClustalW [45,46] and manually screened for redundant sequences, finally resulting in 45 unique WRKY protein coding sequences. The 45 sequences were used as query sequences in a BLASTn search against PlantGDB's EST assemblies (called PUT), in order to obtain maximum sequence length each cDNA from overlapping ESTs [47,48].

A keyword search on NCBI returned 7 mRNA and one genomic sequence of the species *Hordeum vulgare *[GenBank:AJ536667, GenBank:AJ853838 GenBank:AJ853839, GenBank:AJ853840, GenBank:AJ853841, GenBank: AJ853842, GenBank:AY323206, GenBank:AY541586]. Multiple sequence alignment revealed that we had identified an EST representing all of them. To create a uniform nomenclature with constitutive numbering, the sequences annotated as "Hordeum vulgare mRNA for putative WRKY protein" 2 [GenBank:AJ853838] and 5 [GenBank:AJ853841] were named HvWRKY2 and 5, respectively. The GenBank sequence AY541586 ("Hordeum vulgare WRKY transcription factor (wrky38) gene") was published as HvWRKY-38 [31] and is identical with the "Hordeum vulgare mRNA for putative WRKY1 protein" [GenBank:AJ536667]. We named both sequences HvWRKY1 and did not annotate any sequence as "HvWRKY38" to prevent confusion. Sequences "Hordeum vulgare partial mRNA for putative WRKY3 protein" [GenBank:AJ853839] and "Hordeum vulgare partial mRNA for putative WRKY4 protein" [GenBank:AJ853840] showed 100% nucleotide sequence similarity to longer EST sequences which were consequently annotated as HvWRKY3 [GenBank:EF488104] and HvWRKY4 [GenBank:EF488105]. The sequence "Hordeum vulgare partial mRNA for putative WRKY6 protein" [GenBank:AJ853842] had been annotated with a single C-terminal WRKY domain. We extended the sequence to the N-terminal WRKY domain by sequencing and annotated the sequence as HvWRKY6 [GenBank:EF488106]. The sequence "Hordeum vulgare SUSIBA2 (susiba2) mRNA" [GenBank:AY323206] [15] was named HvWRKY46. All remaining identified barley WRKY sequences were annotated on NCBI as HvWRKY7 to -37 and HvWRKY39 to -45 (see Table 1 for accession numbers).

### Phylogenetic analysis

To gain insights into the evolutionary relationships of barley WRKY proteins and WRKY proteins of Arabidopsis and rice (*Oryza sativa*) we performed multiple alignments of the species WRKY proteins. Pairwise alignment using NCBI blastP [47] was used for paraphyletic grouping of HvWRKY36, HvWRKY6 and OsWRKY82 into their respective WRKY groups. Sequence information for 72 Arabidopsis WRKY proteins (AtWRKY) and 81 rice WRKY proteins (OsWRKY [21]) was retrieved from NCBI. WRKY proteins of *Physcomitrella patens *[GenBank:AAL78681, GenBank:ABI64128 GenBank: ABI64129, GenBank: ABI64130, GenBank: ABI64131, GenBank: ABI64132, GenBank: ABI64133, GenBank: ABI64134, GenBank: ABI64135, GenBank: ABI64136], *Giardia lamblia *[GenBank:XM_765980] and *Dictyostelium discoideum *[GenBank:XM_638694] as well as zinc-finger domains of three additional members of the WRKY- GCM1superfamily, namely Arabidopsis Mutator-like transposase [GenBank: AAC97215], *Homo sapiens *CRA a FLYWCH-type zinc finger 1 [GenBank: EAW85450] and *Mus musculus *FLYWCH-type zinc finger 1 [GenBank: AAH38031] were included to form basal outgroups. The 60 amino acid spanning WRKY core domain [16] of all proteins was used to create multiple protein sequence alignments using ClustalW [45,46]. Default settings were applied for the alignment in Figure 1. For Additional Figure 1, default settings were used except for using a PAM matrix, a gap opening penalty of 2 and a gap extension penalty of 0.05. The phylogenetic trees in Figure 2 and Additional File 2 were strict consensus trees of up to 200 000 trees calculated with three different programs, ClustalW [46], Phylip [49] and PAUP [50], which all resulted in identical tree topologies. Bootstrap values have been calculated from 1000 iterations using Phylip [49]. Phylogenetic trees were drawn using TreeView software version 1.6.6 [51]. The tree in Additional Figure 2 was rooted with the zinc finger domain of Arabidopsis mutator-like transposase (AtMutTrans) [GenBank:AAC97215] as outgroup.

### Microarray based expression analysis

For expression analysis, publicly available microarray data of the Affymetrix Barley1 GeneChip were used. The annotations of the Barley1 GeneChip were updated by the HarvEST database [52]. In order to identify probe sets for the barley WRKY genes described in this study, BLAST searches against the Barley1 GeneChip assemblies were performed on the Affymetrix Netaffx website [53]. For 20 of the characterized WRKY genes, probesets could be assigned unambiguously. HvWRKY2 and HvWRKY46 are represented by three and two probesets, respectively, so that a total number of 23 probesets was used to examine the expression of barley WRKY genes. Additional probesets which – according to the HarvEST annotations – have rice or Arabidopsis WRKY genes as best matches were not considered further as they could not be specifically related to any barley EST. The Affymetrix CEL files of powdery mildew treatment experiments (accession numbers BB4 and BB7) as well as the developmental baseline (accession number BB3 [36]) were downloaded from BarleyBase [27,28].

Affymetrix CEL files of the developmental baseline of Arabidopsis [37] were retrieved from TAIR accession number ME00319 [54]. Analysis of microarray data was performed using GeneSpring software version 7 [55].

A per-Chip normalization to the median was applied to obtain comparability. The arrays were adjusted for background optical noise using sequence dependent robust multi-array averaging (GC-RMA) software [56] and normalized using quantile normalization. From the resulting signal intensities, fold change values were calculated.

Expression data were extracted for homologous plant organs (1 – 10) for barley and Arabidopsis, respectively: 1 (coleoptile/cotyledon), 2 (mesocotyl/hypocotyl), 3 (radicule/seedling roots), 4 (leaf/leaf), 5 (root/root), 6 (inflorescence/inflorescence), 7 (anthers/stamen), 8 (caryopsis 5dpa/seed stage 6), 9 (caryopsis 10dpa/seed stage 8) and 10 (caryopsis 15dpa/seed stage 10).

The correlation for gene expression levels between corresponding organs from barley and Arabidopsis were analyzed by calculating the Pearson correlation of the normalized signal intensities with the Microsoft Excel statistical analysis tools. As a control, the signal intensities of 100 randomly chosen probesets of the Barley1 GeneChip were compared to 100 randomly chosen Arabidopsis genes represented by the ATH1 GeneChip. For both comparisons, the average correlation was calculated. Student's t-test was used to obtain the statistical significance of the difference in correlation of the two datasets.

### Plant material

Barley and Arabidopsis (Catania-0) plants were grown in 10 cm pots in soil in the green house (long day; ~24°C; 40% rel. humidity). Rice, *Oryza sativa japonica *cv. Nipponbare, was grown in 60 cm pots in water logged soil in the green house (long day; ~27°C; ~70% rel humidity)

### RNA extraction

Leaf and root plant material from four week old barley and rice plants was harvested at the middle of the day. Infructescences were harvested at the age of eleven days post anthesis. Tissues of Arabidopsis were harvested from three-week-old Catania-0 plants. Plant material was pooled from several plants. Total RNA was isolated according to the RNAqueous system (Ambion; barley) or Trizol (Invitrogen; Arabidopsis and rice) following the manufacturers' recommendations. Plant RNA isolation Aid solution (Ambion) was added to all samples. After extraction, the samples were DNase treated using the DNA-free kit (Ambion). RNA concentration was measured using a NanoDrop ND-1000 (Fisher Scientific).

### RT-PCR

A two-step semi-quantitative RT-PCR method was used to measure gene expression. First strand cDNA synthesis was performed using SuperScript III Reverse Transcriptase (Invitrogen) on 2 – 5 μg total mRNA and oligo dT primers (Invitrogen). The relative amount of gene expression for different WRKY genes was determined within linear amplification ranges. One or two microliters cDNA template (equivalent to 50 and 100 ng RNA) were used for each PCR. Number of cycles used and primer sequences for *WRKY *gene and *Actin *controls are listed in Additional File 6.

Signal intensities of each of the RT-PCR amplicons have been quantified using KODAK Molecular Imaging Systems and Software. Normalized signal intensities have been calculated by dividing the intensity for each *WRKY *gene by the intensity of its respective *Actin *control signal. Normalized fold intensities were calculated for reasons of comparison from the normalized signal intensities divided by the average of the normalized signal intensities of the three organs (mean expression intensity).

### Sequencing Barley WRKY genes

Multiple sequence alignments indicated, that EST sequences BU990739 and BM370186 might possibly represent parts of the C-terminal and the N-terminal WRKY domains of HvWRKY6. To obtain the interjacent sequence, a PCR was performed with HvWRKY6 forward primer 5'-GCATATTCAGAAGGGCTGCCGAG-3' and HvWRKY6 reverse primer 5'-CTGCCCATACTTACGCCATCTG-3'.

Sequence information of the WRKY domain for HvWRKY9 was available (CB879962) and multiple alignments indicated that HvWRKY9 belonged to the subgroup of HARF-domain containing WRKY proteins (for HARF sequence information, see [16]). To extend the sequence further to the N-terminus towards the HARF domain, a PCR was performed with HvWRKY9 forward primer: 5'-AATGGCAGACAAGGCCATGCTAGG-3' and HvWRKY9 reverse primer 5'- ATACTTCCGCCACGAGAATTCA-3'. In a sequencing approach, DNA of barley leaves was isolated using a protocol by Edwards et al. [57,58] and treated with RNase H. PCR was performed with Phusion-Polymerase according to the manufacturer's recommendations (Finnzymes). The PCR products were gel-purified and cloned into a pCR 2.1-TOPO vector (Invitrogen) for sequencing at 4BaseLab (Reutlingen/Germany).

## Authors' contributions

EM and DW carried out the molecular genetic studies. JK compiled expression data and performed all necessary normalization for cross-species comparison. EM and JK carried out the cross-species expression comparison. EM and KWB carried out the statistical analysis and calculation of relative correlation coefficient in randomly chosen and HvWRKY-AtWRKY gene pairs. EM, UHK and DW performed the phylogenetic analysis of WRKY proteins. KH and CJ participated in design and coordination. EM and CH initiated the analysis of barley WRKY genes. EM and DW designed the experiments and wrote the manuscript. All authors read and approved the final manuscript.

## Supplementary Material

Additional File 1 Multiple alignment of WRKY_GCM1-like domains. Multiple alignment of WRKY_GCM1-like domain amino acid sequences from Arabidopsis (AtWRKY), barley (HvWRKY), rice (OsWRKY), Physcomitrella (PpWRKY), *Dictyostelium discoideum *(DdWRKY), *Giardia lamblia *(GlWRKY),*Homo sapiens *(HsFLYWCH) and *Mus musculus *(MmFLYWCH). N-terminal and C-terminal WRKY domains of group 1 WRKY proteins are indicated as _N and _C, respectively. The conserved WRKY signature is highlighted in bold letters, the amino acids forming the zinc-finger motif are shaded with grey, gaps in the alignment are indicated by dashes. Click here for file

Additional File 2 Phylogenetic tree based on all WRKY_GCM1-like domains considered in the Alignment of Additional File 1. Phylogenetic tree based on the WRKY domain amino acid sequences given in Additional Figure 1. Sequences of Arabidopsis (AtWRKY), barley (HvWRKY), Physcomitrella (PpWRKY), rice (OsWRKY), *Dictyostelium discoideum *(DdWRKY), *Giardia lamblia *(GlWRKY) *Homo sapiens *(HsFLYWCH) and *Mus musculus *(MmFLYWCH) are arranged in clusters with the zinc finger domain of the Arabidopsis mutator-like transposase (AtMutTrans) as outgroup. Tree topology has been confirmed by using three different programs. Bootstrap values from 1000 iterations and bigger than 500 are included in the tree. HvWRKY sequences are highlighted in bold letters. Groups and subgoups of WRKY1 to 3 are indicated by bars on the right side. Click here for file

Additional File 3 Normalized signal intensities of *HvWRKY *genes, *AtWRKY *genes and a set of 101 randomly chosen gene pairs. The table contains all essential data for the comparative gene expression analysis for barley and Arabidopsis *WRKY *genes as well as control gene pairs on the extracted subsets of the microarray expression datasets of homologous organs by Druka et al. [36] and Schmid et al. [37]. Click here for file

Additional File 4 Signal intensities of RT-PCR experiment with barley, Arabidopsis and rice *WRKY *genes. Tables contain raw signal intensity values of bands from RT-PCR experiments shown in Figure 5. Click here for file

Additional File 5 Distribution of the relative correlation coefficient in randomly chosen and *HvWRKY-AtWRKY *gene pairs. The relative correlation coefficient was calculated from the normalized signal intensities of the gene pairs and plotted according to its occurrence. Random gene pairs exhibited an average correlation coefficient of 0.01. WRKY gene pairs exhibited a divided curve with two maxima. When only the best pairing orthologs were considered, the first peak of more random distribution is lost. Click here for file

Additional File 6 Primer sequences used in RT-PCR experiment with barley, Arabidopsis and rice *WRKY *genes. Table containing the sequences of primers, numbers of cycles and expected fragment size of RT-PCR experiments depicted in Figure 5. Click here for file
