# Supplementary material for: Phylogenetic and comparative gene expression analysis of barley (Hordeum vulgare) WRKY transcription factor family reveals putatively retained functions between monocots and dicots
Source: BMC Genomics. 2008 Apr 28;9:194. doi: 10.1186/1471-2164-9-194 (PMC2390551; doi:10.1186/1471-2164-9-194)
Supplement: Additional File 1 — Multiple alignment of WRKY_GCM1-like domains. Multiple alignment of WRKY_GCM1-like domain amino acid sequences from Arabidopsis (AtWRKY), barley (HvWRKY), rice (OsWRKY), Physcomitrella (PpWRKY), Dictyostelium discoideum (DdWRKY), Giardia lamblia (GlWRKY),Homo sapiens (HsFLYWCH) and Mus musculus (MmFLYWCH). N-terminal and C-terminal WRKY domains of group 1 WRKY proteins are indicated as _N and _C, respectively. The conserved WRKY signature is highlighted in bold letters, the amino acids forming the zinc-finger motif are shaded with grey, gaps in the alignment are indicated by dashes. [file 1471-2164-9-194-S1.doc]

**GROUP1**

OsWRKY24_N SQRRSSDDGYN**WRKY**GQKQ-VKGSENP--------------------------RSYYKCTFPN--------CP-------------TKKKVERSL-D-------GQI---------------TEIVYKG--------THNHAKP

AtWRKY33_N REQRKGEDGYN**WRKY**GQKQ-VKGSENP--------------------------RSYYKCTFPN--------CP-------------TKKKVERSL-E-------GQI---------------TEIVYKG--------SHNHPKP

AtWRKY26_N SSNKTSDDGYN**WRKY**GQKQ-VKGSENP--------------------------RSYFKCTYPN--------CL-------------TKKKVETSLVK-------GQM---------------IEIVYKG--------SHNHPKP

OsWRKY70_N QSRRSSDDGYN**WRKY**GQKQ-MKGSENP--------------------------RSYYKCTFPG--------CP-------------TKKKVEQSP-D-------GQV---------------TEIVYKG--------AHSHPKP

AtWRKY25_N MVSRNSNDGYG**WRKY**GQKQ-VKKSENP--------------------------RSYFKCTYPD--------CV-------------SKKIVETAS-D-------GQI---------------TEIIYKG--------GHNHPKP

OsWRKY57 AKNSLSYDGYS**WRKY**GQKQ-VKGSEFP--------------------------RSYYKCTHPT--------CP-------------VKRKVEMTP-D-------GRI---------------AEIVYNG--------EHNHPKP

HvWRKY40-N -----------**----**GTR------EFP--------------------------RSYYKCTHPT--------CP-------------VKRKVETTV-D-------GQI---------------AEIVYNG--------EHNHPQP

AtWRKY44_N TGDRSSVDGYN**WRKY**GQKQ-VKGSECP--------------------------RSYYKCTHPK--------CP-------------VKKKVERSV-E-------GQV---------------SEIVYQG--------EHNHSKP

HvWRKY42_N TNSNPGEDGYN**WRKY**GQKQ-VKSSEHP--------------------------RSYYKCTHPD--------CP-------------VKKKVERSQ-D-------GQI---------------TEIVYKS--------SHNHPLP

OsWRKY53_N GGNNKLEDGYN**WRKY**GQKQ-VKGSENP--------------------------RSYYKCTYNG--------CS-------------MKKKVERSLAD-------GRI---------------TQIVYKG--------AHNHPKP

AtWRKY2_N AGGAPAEDGYN**WRKY**GQKL-VKGSEYP--------------------------RSYYKCTNPN--------CQ-------------VKKKVERSR-E-------GHI---------------TEIIYKG--------AHNHLKP

AtWRKY34_N ACCAPADDGYN**WRKY**GQKL-VKGSEYP--------------------------RSYYKCTHPN--------CE-------------AKKKVERSR-E-------GHI---------------IEIIYTG--------DHIHSKP

OsWRKY35_N AAAAVAEDGYS**WRKY**GQKQ-VKHSEYP--------------------------RSYYKCTHAS--------CA-------------VKKKVERSH-E-------GHV---------------TEIIYKG--------THNHPKP

OsWRKY78_N VAEKSAEDGYN**WRKY**GQKH-VKGSENP--------------------------RSYYKCTHPN--------CD-------------VKKLLERSL-D-------GQI---------------TEVVYKG--------RHNHPKP

hvWRKY46_N VADKSADDGYN**WRKY**GQKH-VKGSENP--------------------------RSYYKCTHPN--------CE-------------VKKLLERAV-D-------GLI---------------TEVVYKG--------RHNHPKP

AtWRKY20_N TPSILADDGYN**WRKY**GQKH-VKGSEFP--------------------------RSYYKCTHPN--------CE-------------VKKLFERSH-D-------GQI---------------TDIIYKG--------THDHPKP

AtWRKY3_N NADKPADDGYN**WRKY**GQKQ-VKGSDFP--------------------------RSYYKCTHPA--------CP-------------VKKKVERSL-D-------GQV---------------TEIIYKG--------QHNHELP

AtWRKY4_N NVDKPADDGYN**WRKY**GQKQ-VKGSEFP--------------------------RSYYKCTNPG--------CP-------------VKKKVERSL-D-------GQV---------------TEIIYKG--------QHNHEPP

AtWRKY19_N NVDKQVNDGYN**WQKY**GQKK-VKGSKFP--------------------------LSYYKCTYLG--------CP-------------SKRKVERSL-D-------GQV---------------AEIVYKD--------RHNHEPP

AtWRKY58_N NVDKPADDGYN**WRKY**GQKP-IKGCEYP--------------------------RSYYKCTHVN--------CP-------------VKKKVERSS-D-------GQI---------------TQIIYKG--------QHDHERP

OsWRKY4_N AIEQPAKDGYN**WRKY**GQKQ-LKDAESP--------------------------RSYYKCTRDG--------CP-------------VKKIVERSS-D-------GCI---------------KEITYKG--------RHSHPRP

OsWRKY82_N VSVNMVGDGFN**WRKY**GQKQ-VKSSENS--------------------------RSYYRCTNSN--------CL-------------AKKKVEHCPD--------GRV---------------VEIIYRG--------THNHEPP

HvWRKY6-N AAINIVGDGFN**WRKY**GQKQ-VKSSDNS--------------------------RSYYRCTNSS--------CL-------------AKKKVEHCPD--------GRI---------------IEIIYRG--------THSHEPP

AtWRKY32_N VPRTPARDGYN**WRKY**GQKQ-VKSPKGS--------------------------RSYYRCTYTE--------CC-------------AKK-IECSNDS-------GNV---------------VEIVNKG--------LHTHEPP

HvWRKY41_N ---ARGDDGIN**WRKY**GQKA-VKGGKCP--------------------------RSYYKCT-LN--------CP-------------VRKNVEHSAD--------GRI---------------IKIVYRG--------QHCHEPP

AtWRKY1_N IREKVMEDGYN**WRKY**GQKL-VKGNEFV--------------------------RSYYRCTHPN--------CK-------------AKKQLERSA-G-------GQV---------------VDTVYFG--------EHDHPKP

OsWRKY24_C SDIDILDDGYR**WRKY**GQKV-VKGNPNP--------------------------RSYYKCTTAG--------CP-------------VRKHVERASHD-------LRA---------------VITTYEG--------KHNHDVP

OsWRKY70_C SDIDILDDGYR**WRKY**GQKV-VKGNPNP--------------------------RSYYKCTTAG--------CP-------------VRKHVERASND-------LRA---------------VITTYEG--------KHNHDVP

AtWRKY33_C SDIDILDDGYR**WRKY**GQKV-VKGNPNP--------------------------RSYYKCTTIG--------CP-------------VRKHVERASHD-------MRA---------------VITTYEG--------KHNHDVP

OsWRKY53_C SDIDILDDGFR**WRKY**GQKV-VKGNPNP--------------------------RSYYKCTTVG--------CP-------------VRKHVERASHD-------TRA---------------VITTYEG--------KHNHDVP

HvWRKY43 SDIDILDDGYR**WRKY**GQKV-VKGNPNP--------------------------RSYYKCTTPN--------CP-------------VRKHVERASQD-------LRA---------------VVTTYEG--------KHNHDVP

OsWRKY30 SEVDILDDGYR**WRKY**GQKV-VKGNPNP--------------------------RSYYKCTHPG--------CS-------------VRKHVERSSHD-------LKS---------------VITTYEG--------KHNHEVP

OsWRKY35_C SEVDILDDGYR**WRKY**GQKV-VKGNPNPSSSSSMDADRSLVVVVVI--------RSYYKCTHPG--------CL-------------VRKHVERASHD-------LKS---------------VITTYEG--------KHNHEVP

OsWRKY80 SEVDILDDGYR**WRKY**GQKV-VKGNPNP--------------------------RSYYKCTHQG--------CS-------------VRKHVERASHD-------LKS---------------VITTYEG--------KHNHEVP

HvWRKY42_C SEVDILDDGYR**WRKY**GQKV-VKGNPNP--------------------------RSYYKCTHQG--------CS-------------VRKHVERASHD-------LKS---------------VITTYXG--------KHNHEVP

AtWRKY2_C SDVDILDDGYR**WRKY**GQKV-VKGNPNP--------------------------RSYYKCTAPG--------CT-------------VRKHVERASHD-------LKS---------------VITTYEG--------KHNHDVP

OsWRKY78_C SEVDILDDGYR**WRKY**GQKV-VKGNPNP--------------------------RSYYKCTNTG--------CP-------------VRKHVERASHD-------PKS---------------VITTYEG--------KHNHEVP

HvWRKY46_C SEVDILDDGYR**WRKY**GQKV-VKGNPNP--------------------------RSYYKCTSTG--------CP-------------VRKHVERASHD-------PKS---------------VITTYEG--------KHNHEVP

AtWRKY20_C SEVDILDDGYR**WRKY**GQKV-VRGNPNP--------------------------RSYYKCTAHG--------CP-------------VRKHVERASHD-------PKA---------------VITTYEG--------KHDHDVP

PpWRKY4 SDVDILDDGYR**WRKY**GQKV-VKGNPHP--------------------------RSYYKCTNVG--------CP-------------VRKHVERASND-------PKA---------------VITTYEG--------KHNHDVP

AtWRKY3_C SEVDLLDDGYR**WRKY**GQKV-VKGNPYP--------------------------RSYYKCTTPD--------CG-------------VRKHVERAATD-------PKA---------------VVTTYEG--------KHNHDVP

AtWRKY4_C SEVDLLDDGYR**WRKY**GQKV-VKGNPYP--------------------------RSYYKCTTPG--------CG-------------VRKHVERAATD-------PKA---------------VVTTYEG--------KHNHDLP

AtWRKY58_C SEVDLLDDGYR**WRKY**GQKV-VKGNPHP--------------------------RSYYKCTTPN--------CT-------------VRKHVERASTD-------AKA---------------VITTYEG--------KHNHDVP

AtWRKY26_C SDIDILDDGYR**WRKY**GQKV-VKGNPNP--------------------------RSYYKCTFTG--------CF-------------VRKHVERAFQD-------PKS---------------VITTYEG--------KHKHQIP

AtWRKY34_C SDIDILDDGYR**WRKY**GQKV-VKGNPNP--------------------------RSYYKCTANG--------CT-------------VTKHVERASDD-------FKS---------------VLTTYIG--------KHTHVVP

HvWRKY41_C SDVDLLDDGYR**WRKY**GQKV-VRGNPHP--------------------------RSYYKCTYQG--------CD-------------VKKHVERSSQE-------PHA---------------VITTYEG--------KHTHDVP

OsWRKY4_C SEVDLLDDGYR**WRKY**GQKV-VKGNPRP--------------------------RSYYKCTADG--------CN-------------VRKQIERASAD-------PKC---------------VLTTYTG--------RHNHDPP

AtWRKY25_C SDIDVLIDGFR**WRKY**GQKV-VKGNTNP--------------------------RSYYKCTFQG--------CG-------------VKKQVERSAAD-------ERA---------------VLTTYEG--------RHNHDIP

AtWRKY10 SDEDNPNDGYR**WRKY**GQKV-VKGNPNP--------------------------RSYFKCTNIE--------CR-------------VKKHVERGADN-------IKL---------------VVTTYDG--------IHNHPSP

OsWRKY33 VNIDILDAGFR**WRKY**GQKV-VKGNPNP-----------------------SRGTTPSRCSPIP---PAFTAANG------------AGAAFQRTKDK-------PRD---------------DLFVES-------------LLC

OsWRKY38 INIDILDAGFR**WRKY**GQKV-VKGNPNPRSYYKCTTVGCPVRKHVERALHDTRAVITTYAGAVVQRDPAVGSANG------------AGAAFQRTKDK-------PRD---------------DLFVES-------------LLC

HvWRKY40-C ECESSRDAAFR**WRKY**GQKA-VNGNSFP--------------------------RSYYRCSTAR--------CN------------------ARK--------------------------------------------------

AtWRKY44_C VESDSLEDGFR**WRKY**GQKV-VGGNAYP--------------------------RSYYRCTSAN--------CR-------------ARKHVERASDD-------PRA---------------FITTYEG--------KHNHHLL

OsWRKY82_C VQAGKTSDGYR**WRKY**GQKI-VKGNPNP--------------------------RSYYRCTHDG--------CP-------------VRKHVEKAPDD-------DNN---------------IVVTYEG--------KHNHDQP

HvWRKY6_C RAAAKMSDGYR**WRKY**GQKI-VKGNPNP--------------------------RSYYRCTHDG--------CP-------------VRKHVERAPDD-------INN---------------MVVTYEG--------KHNHGQP

AtWRKY1_C TLFDIVNDGYR**WRKY**GQKS-VKGSPYP--------------------------RSYYRCSSPG--------CP-------------VKKHVERSSHD-------TKL---------------LITTYEG--------KHDHDMP

AtWRKY32_C GDVGICGDGYR**WRKY**GQKM-VKGNPHP--------------------------RNYYRCTSAG--------CP-------------VRKHIETAVEN-------TKA---------------VIITYKG--------VHNHDMP

AtWRKY19_C SEVDNLDDGYR**WRKY**GQKV-VKGNPYP--------------------------RFSSSKDYDVVI-----RYGRADISNEDFISHLRASLCRRGISVY------EKFNEVDALPKCRVLIIVLTSTYVP-SNLLNILEHQHTED

HvWRKY35_C SDVDLLDDGYR**WRKY**GQKV-ERGNPYP--------------------------GHGT---FFVLQ-----QC-------------MKKALHSHSREIY------T---------------------------------------

**Group2a**

OsWRKY28 DLSLVVKDGYQ**WRKY**GQKV-TKDNPCP--------------------------RAYFRCSFAPA-------CP-------------VKKKVQRSADD-------NTV---------------LVATYEG--------EHNHAQP

OsWRKY71 DLSLVVKDGYQ**WRKY**GQKV-TKDNPCP--------------------------RAYFRCSFAPA-------CP-------------VKKKVQRSAED-------NTI---------------LVATYEG--------EHNHGQP

HvWRKY1 DLSLVVKDGYQ**WRKY**GQKV-TKDNPCP--------------------------RAYFRCSFAPG-------CP-------------VKKKVQRSAED-------KTI---------------LVATYEG--------EHNHTQP

HvWRKY2 DLSLVVKDGYQ**WRKY**GQKV-TKDNPCP--------------------------RAYFRCSSAPS-------CQ-------------VKKKVQRSAED-------KTV---------------LVATYDG--------DHNHAPP

HvWRKY23 DLSLVVKDGYQ**WRKY**GQKV-TKDNPCP--------------------------RAYFRCSFAPS-------CQ-------------VKKKVQRSAED-------KTV---------------LVATYDG--------DHNHAPP

OsWRKY76 DTSLVVKDGYQ**WRKY**GQKV-TRDNPSP--------------------------RAYFRCAFAPS-------CP-------------VKKKVQRSAED-------SSL---------------LVATYEG--------EHNHPHP

HvWRKY3 DTSLVVKDGYQ**WRKY**GQKV-TRDNPSP--------------------------RAYFRCAFAPS-------CP-------------VKKKVQRSAED-------SSM---------------VEATYEG--------EHNHPRP

AtWRKY40 DTTLVVKDGYQ**WRKY**GQKV-TRDNPSP--------------------------RAYFKCACAPS-------CS-------------VKKKVQRSVED-------QSV---------------LVATYEG--------EHNHPMP

AtWRKY18 VTLQTVKDGFQ**WRKY**GQKV-TRDNPSP--------------------------RAYFRCSFAPS-------CP-------------VKKKVQRSAED-------PSL---------------LVATYEG--------THNHLGP

AtWRKY60 DTSLTVKDGYQ**WRKY**GQKI-TRDNPSP--------------------------RAYFRCSFSPS-------CL-------------VKKKVQRSAED-------PSF---------------LVATYEG--------THNHTGP

OsWRKY62 SMAETVKDGYQ**WRKY**GQKV-TRDNPYP--------------------------RAYFRCAFAPS-------CP-------------VKKKLQRCAED-------RSM---------------LVATYEG--------EHNHALS

**Group2b**

AtWRKY6 SEAPMISDGCQ**WRKY**GQKM-AKGNPCP--------------------------RAYYRCTMATG-------CP-------------VRKQVQRCAED-------RSI---------------LITTYEG--------NHNHPLP

AtWRKY31 SEAAMISDGCQ**WRKY**GQKM-AKGNPCP--------------------------RAYYRCTMAGG-------CP-------------VRKQVQRCAED-------RSI---------------LITTYEG--------NHNHPLP

AtWRKY42 SEAPMLSDGCQ**WRKY**GQKM-AKGNPCP--------------------------RAYYRCTMAVG-------CP-------------VRKQVQRCAED-------RTI---------------LITTYEG--------NHNHPLP

OsWRKY43 SDAPMISDGCQ**WRKY**GQKM-AKGNPCP--------------------------RAYYRCTMAAG-------CP-------------VRKQVQRCAED-------RTV---------------LITTYEG--------NHNHPLP

OsWRKY1 SEAPIIADGCQ**WRKY**GQKM-AKGNPCP--------------------------RAYYRCTMATG-------CP-------------VRKQVQRCAED-------RSI---------------LITTYEG--------THNHPLP

OsWRKY5 SEAPMISDGCQ**WRKY**GQKM-AKGNPCP--------------------------RAYYRCTMASQ-------CP-------------VRKQVQRCAKD-------KSI---------------LITTYEG--------THSHPLP

AtWRKY47 SDATTVNDGCQ**WRKY**GQKM-AKGNPCP--------------------------RAYYRCTMAVG-------CP-------------VRKQVQRCAED-------TTI---------------LTTTYEG--------NHNHPLP

OsWRKY9 KPRVSISDGCQ**WRKY**GQKM-AKGNPCP--------------------------RAYYRCTMAIG-------CP-------------VRKQVQRCAED-------KTV---------------LITTYEG--------NHNHQLP

OsWRKY32 CQGPTMNDGCQ**WRKY**GQKV-AKGNPCP--------------------------RAYYRCTVAPG-------CP-------------VRKQVQRCLED-------MSI---------------LVTTYEG--------THNHPLP

AtWRKY9 CETATMNDGCQ**WRKY**GQKT-AKGNPCP--------------------------RAYYRCTVAPG-------CP-------------VRKQVQRCLED-------MSI---------------LITTYEG--------THNHPLP

AtWRKY72 CDTPTMNDGCQ**WRKY**GQKI-AKGNPCP--------------------------RAYYRCTVAPG-------CP-------------VRKQVQRCADD-------MSI---------------LITTYEG--------THSHSLP

HvWRKY37 CDTPTMNDGCQ**WRKY**GQKI-SKGNPCP--------------------------RAYYRCTVAPS-------CP-------------VRKQVQRCADD-------MSI---------------LITTYEG--------THSHPLP

OsWRKY73 CDAPTMNDGCQ**WRKY**GQKI-AKGNPCP--------------------------RAYYRCTVAAG-------CP-------------VRKQVQRCADD-------MSI---------------LITTYEG--------THNHPL-

AtWRKY61 KTRVSMNDGCQ**WRKY**GQKI-AKGNPCP--------------------------RAYYRCTIAAS-------CP-------------VRKQVQRCSED-------MSI---------------LISTYEG--------THNHPLP

PpWRKY5 -------DGCQ**WRKY**GQKM-AKGNPWP--------------------------RAYFRCTVSPG-------CP-------------VRKQVQRCEED-------TSI---------------LVTTYEG--------THNHALS

AtWRKY36 CEDPSINDGCQ**WRKY**GQKT-AKTNPLP--------------------------RAYYRCSMSSN-------CP-------------VRKQVQRCGEEE------TSA---------------FMTTYEG--------NHDHPLP

OsWRKY27 CSAPTVKDGCQ**WRKY**GQKT-AKGNPWP--------------------------RGYYRCTGAPG-------CP-------------VKKQVQRCNHD-------TSV---------------LVTTYDG--------VHNHPIT

**Group2c**

OsWRKY3 SEIDHLEDGYR**WRKY**GQKA-VKNSPFP--------------------------RSYYRCTNSK--------CT-------------VKKRVERSSDD-------PSV---------------VITTYEG--------QHCHHTA

OsWRKY29 SEIDHLEDGYR**WRKY**GQKA-VKNSPFP--------------------------RSYYRCTNSK--------CT-------------VKKRVERSSDD-------PSV---------------VITTYEG--------QHSHHTV

HvWRKY15 SEVDHLEDGYR**WRKY**GQKA-VKNSPFP--------------------------RSYYRCTNSK--------CT-------------VKKRVERSSED-------P---------------------------------------

AtWRKY57 SDVDNLEDGYR**WRKY**GQKA-VKNSPFP--------------------------RSYYRCTNSR--------CT-------------VKKRVERSSDD-------PSI---------------VITTYEG--------QHCHQTI

OsWRKY8 SEVDHLEDGYR**WRKY**GQKA-VKNSSYP--------------------------RSYYRCTAPR--------CG-------------VKKRVERSEQD-------PSM---------------VITTYEG--------QHTHPSP

OsWRKY11 SEVDHLEDGYR**WRKY**GQKA-VKNSPYP--------------------------RSYYRCTTPK--------CG-------------VKKRVERSYQD-------PST---------------VITTYEG--------QHTHHSP

AtWRKY23 SEVDHLEDGYR**WRKY**GQKA-VKNSPFP--------------------------RSYYRCTTAS--------CN-------------VKKRVERSFRD-------PST---------------VVTTYEG--------QHTHISP

OsWRKY16 SEVDHLEDGYR**WRKY**GQKA-VKNSPYP--------------------------RSYYRCTTQK--------CP-------------VKKRVERSYQD-------PAV---------------VITTYEG--------KHTHPIP

OsWRKY49 SEVDHLEDGYR**WRKY**GQKA-VKNSPFP--------------------------RSYYRCTTQK--------CP-------------VKKRVERSYQD-------AAV---------------VITTYEG--------KHTHPIP

AtWRKY8 TEVDHLEDGYR**WRKY**GQKA-VKNSPYP--------------------------RSYYRCTTQK--------CN-------------VKKRVERSYQD-------PTV---------------VITTYES--------QHNHPIP

AtWRKY28 SEVDHLEDGYR**WRKY**GQKA-VKNSPYPRIIANGNEN-----------------RSYYRCTTQK--------CN-------------VKKRVERSFQD-------PTV---------------VITTYEG--------QHNH---

AtWRKY71 SEIDHLEDGYR**WRKY**GQKA-VKNSPYP--------------------------RSYYRCTTQK--------CN-------------VKKRVERSFQD-------PSI---------------VITTYEG--------KHNHPIP

AtWRKY48 SDIDNLDDGYR**WRKY**GQKA-VKNSPYP--------------------------RSYYRCTTVG--------CG-------------VKKRVERSSDD-------PSI---------------VMTTYEG--------QHTHPFP

AtWRKY68 SEVLHLDDGYK**WRKY**GQKP-VKDSPFP--------------------------RNYYRCTTTW--------CD-------------VKKRVERSFSD-------PSS---------------VITTYEG--------QHTHPRP

HvWRKY19 TEIEILDDGYK**WRKY**GKKS-VKNSPNP--------------------------RNYYRCSTEG--------CS-------------VKKRVERDRDD-------PAY---------------VVTTYEG--------THSHASP

HvWRKY20 SEIEILDDGYK**WRKY**GKKS-VKNSPNP--------------------------RNYYRCSTEG--------CD-------------VKKRVERDRDD-------PAY---------------VVTTYEG--------THSHASP

OsWRKY7 SEIEILDDGYK**WRKY**GKKS-VKNSPNP--------------------------RNYYRCSTEG--------CN-------------VKKRVERDKDD-------PSY---------------VVTTYEG--------THNHVSP

HvWRKY18 SEEEILDDGYK**WRKY**GKKS-VKNSPNP--------------------------RNYYRCSTEG--------CN-------------VKKRVERDKDD-------AXF---------------VVTMYEG--------VHNHAS-

OsWRKY26 SDDEILDDGYK**WRKY**GKKS-VKNSPNP--------------------------RNYYRCSTEG--------CN-------------VKKRVERDKND-------PRY---------------VVTMYEG--------IHNHVCP

OsWRKY59 SDDEILDDGYK**WRKY**GKKS-VKNSPNP--------------------------RNYYRCSTEG--------CN-------------VKKRVERDKND-------PRY---------------VVTTYEG--------IHNHVCP

OsWRKY10 SEVEVLDDGFK**WRKY**GKKA-VKSSPNP--------------------------RNYYRCSAAG--------CG-------------VKKRVERDGDD-------PRY---------------VVTTYDG--------VHNHATP

HvWRKY17 SEVEILDDGFK**WRKY**GKKA-VKNSPNP--------------------------RNYYRCSAEG--------CG-------------VKKRVERDRDD-------PRY---------------VVTTYDG--------VHNHATP

OsWRKY67 SEVEILDDGFK**WRKY**GKKA-VKNSPNP--------------------------RNYYRCSTEG--------CN-------------VKKRVERDRED-------HRY---------------VITTYDG--------VHNHASP

HvWRKY16 SEVEILDDGFK**WRKY**GKKA-VKNSPNP--------------------------RNYYRCSTEG--------CN-------------VKKRVERDRED-------HRY---------------VITTYDG--------VHTNPLP

OsWRKY77 SEVDVLDDGYR**WRKY**GKKM-VKNSPNP--------------------------RNYYRCSSEG--------CR-------------VKKRVERARDD-------ARF---------------VVTTYDG--------VHNHPAP

HvWRKY5 SEVEVLDDGYR**WRKY**GKKM-VKNSPNP--------------------------RNYYRCSSEG--------CR-------------VKKRVERDRDD-------ERF---------------VITTYDG--------VHNHLAP

AtWRKY50 SEVEVLDDGFK**WRKY**GKKM-VKNSPHP--------------------------RNYYKCSVDG--------CP-------------VKKRVERDRDD-------PSF---------------VITTYEG--------SHNHSSM

AtWRKY51 SKIDVMDDGFKWRKYGKKS-VKNNINK--------------------------RNYYKCSSEG--------CS-------------VKKRVERDGDD-------AAY---------------VITTYEG--------VHNHESL

AtWRKY59 DEKVALDDGYK**WRKY**GKKP-ITGSPFP--------------------------RHYHKCSSPD--------CN-------------VKKKIERDTNN-------PDY---------------ILTTYEG--------RHNHPSP

OsWRKY36 SDVDVLDDGYK**WRKY**GQKV-VKNSLHP--------------------------RSYYRCTHNN--------CR-------------VKKRVERLSED-------CRM---------------VITTYEG--------RHTHTPC

AtWRKY12 SDVDVLDDGYK**WRKY**GQKV-VKNSLHP--------------------------RSYYRCTHNN--------CR-------------VKKRVERLSED-------CRM---------------VITTYEG--------RHNHIPS

OsWRKY34 SEVDVLDDGYK**WRKY**GQKV-VKNSLHP--------------------------RSYFRCTHSN--------CR-------------VKKRVERLSTD-------CRM---------------VITTYEG--------RHTHSPC

OsWRKY79 SDVDVLDDGYK**WRKY**GQKV-VKNTQHP--------------------------RSYYRCTQDN--------CR-------------VKKRVERLAED-------PRM---------------VITTYEG--------RHVHSPS

HvWRKY14 SDVDVLDDGYK**WRKY**GQKV-VKNTQHP--------------------------RSYYRCTQDK--------CR-------------VKKRVERLAED-------PRM---------------VITTYEG--------RHVHSPS

AtWRKY13 SEVDVLDDGYR**WRKY**GQKV-VKNTQHP--------------------------RSYYRCTQDK--------CR-------------VKKRVERLADD-------PRM---------------VITTYEG--------RHLHSPS

PpWRKY6 SEVDVIDDGYK**WRKY**GQKP-VKSSPHP--------------------------RNYYRCTTAN--------CP-------------VRKRVERSIED-------PGL---------------IVTSYEG--------THTHPKI

PpWRKY7 SEVDVIDDGYK**WRKY**GQKP-VKSSPHP--------------------------RNYYRCTTAN--------CP-------------VRKRVERSIED-------PGL---------------IVTSYEG--------THTHPKI

PpWRKY3 SEIDVMEDGYK**WRKY**GQKA-VKDSPFP--------------------------RSYYRCTNQT--------CP-------------VRKRVERKAGD-------AGL---------------VVTTYEG--------THSHLSP

OsWRKY23 SDNDILDDGYR**WRKY**GQKA-VKNSKHP--------------------------RSYYRCTHHT--------CN-------------VKKQVQRLAKD-------TSI---------------VVTTYEG--------VHNHPCE

HvWRKY13 SENDVLDDGYR**WRKY**GQKA-VKNSAFP--------------------------RSYYRCTHHT--------CN-------------VKKQVQRLAKD-------TSI---------------VVTTYEG--------VHNHPCE

AtWRKY24 SDDDVLDDGYR**WRKY**GQKS-VKHNAHP--------------------------RSYYRCTYHT--------CN-------------VKKQVQRLAKD-------PNV---------------VVTTYEG--------VHNHPCE

AtWRKY56 SDDDVLDDGYR**WRKY**GQKS-VKNNAHP--------------------------RSYYRCTYHT--------CN-------------VKKQVQRLAKD-------PNV---------------VVTTYEG--------VHNHPCE

AtWRKY43 SDADILDDGYR**WRKY**GQKS-VKNSLYP--------------------------RSYYRCTQHM--------CN-------------VKKQVQRLSKE-------TSI---------------VETTYEG--------IHNHPCE

OsWRKY72 SQVDILDDGYR**WRKY**GQKA-VKNNKFP--------------------------RSYYRCTHQG--------CN-------------VKKQVQRLSRD-------ETV---------------VVTTYEG--------THTHPIE

HvWRKY12 SQVDILDDGYR**WRKY**GQKA-VKNNNFP--------------------------RSYYRCTHQG--------CN-------------VKKQVQRLSRD-------EGV---------------VVTTYEG--------THTHPIE

AtWRKY75 SQVDILDDGYR**WRKY**GQKA-VKNNKFP--------------------------RSYYRCTYGG--------CN-------------VKKQVQRLTVD-------QEV---------------VVTTYEG--------VHSHPIE

AtWRKY45 SQVDILDDGYR**WRKY**GQKA-VKNNPFP--------------------------RSYYKCTEEG--------CR-------------VKKQVQRQWGD-------EGV---------------VVTTYQG--------VHTHAVD

OsWRKY17 NNGGLADDGYK**WRKY**GQKS-IKNSPNP--------------------------RSYYRCTNP--------RCN-------------AKKQVERAVDEP------D----------------TLIVTYEG--------LHLHYTY

AtWRKY49 NSNGMCDDGYK**WRKY**GQKS-IKNSPNP--------------------------RSYYKCTNP--------ICN-------------AKKQVERSIDES------N----------------TYIITYEG--------FHFHYTY

HvWRKY36 GGKKTPMDGYR**WRKY**GQKF-IKNNPHP--------------------------RSYYKCTSA--------RCS-------------AKKHVEKSTDDP------E----------------MLIVTYEG--------SHLHGPQ

OsWRKY60 VQLVMPEDGYE**WKKY**GQKF-IKNIQKN--------------------------RSYFRCRDQ--------RCG-------------AKKKVEWHPHDP------G---------------LNLRVVYDG--------AHHHGSP

OsWRKY58 SENDILDDGYR**WRKY**GQKA-VKNSDFP--------------------------SDDE--LLFS-------DVD-------------NTQTATENLRFI------P-----------------LGRVYIT--------G------

OsWRKY56 CGGKMPADGYK**WRKY**GQKS-IKNNPHP--------------------------RCAT------------------------------RSIIDPI--------------------------------------------------

**Group2d**

OsWRKY6 KVADIPSDNYS**WRKY**GQKP-IKGSPHPRG--------------------------YYRCSS---K----KDCP-------------ARKHVERCRS--------DPA--------------MLLVTYEN--------EHNHAQP

HvWRKY11 KVADIPSDNYS**WRKY**GQKP-IKGSPHPRG--------------------------YYRCSS---I----KDCP-------------ARKHVERCRG--------DAG--------------MLIVTYEN--------DHNHAQP

OsWRKY68 KIADIPPDEYS**WRKY**GQKP-IKGSPYPRG--------------------------YYKCST---V----RGCP-------------ARKHVERATD--------DPA--------------MLVVTYEG--------EHRHTPG

HvWRKY7 KIADIPPDEYS**WRKY**GQKP-IKGSPYPRG--------------------------YYKCST---V----RGCP-------------ARKHVERALD--------DPA--------------MLVVTYEG--------EHRHSPG

AtWRKY11 KIADIPPDEYS**WRKY**GQKP-IKGSPHPRG--------------------------YYKCST---F----RGCP-------------ARKHVERALD--------DPA--------------MLIVTYEG--------EHRHNQS

AtWRKY17 KIADIPPDEYS**WRKY**GQKP-IKGSPHPRG--------------------------YYKCST---F----RGCP-------------ARKHVERALD--------DST--------------MLIVTYEG--------EHRHHQS

AtWRKY39 KIADIPPDEYS**WRKY**GQKP-IKGSPHPRG--------------------------YYKCSS---V----RGCP-------------ARKHVERCID--------ETS--------------MLIVTYEG--------EHNHSRI

AtWRKY74 KIADIPPDEYS**WRKY**GQKP-IKGSPHPRG--------------------------YYKCSS---V----RGCP-------------ARKHVERCVE--------ETS--------------MLIVTYEG--------EHNHSRI

HvWRKY8 KISDIPPDEYS**WRKY**GQKP-IKGSPHPRG--------------------------YYKCST---V----RGCP-------------ARKHVERCVD--------EPA--------------MLIVTYEG--------EHSHNRL

AtWRKY7 KMADIPSDEFS**WRKY**GQKP-IKGSPHPRG--------------------------YYKCSS---V----RGCP-------------ARKHVERALD--------DAM--------------MLIVTYEG--------DHNHALV

HvWRKY9 KVADIPADEFS**WRKY**GQKP-IKGSPHPRG--------------------------YYKCSS---V----RGCP-------------ARKHVERCVD--------DPA--------------MLIVTYEG--------DHNHNRA

AtWRKY15 KMSDVPPDDYS**WRKY**GQKP-IKGSPHPRG--------------------------YYKCSS---V----RGCP-------------ARKHVERAAD--------DSS--------------MLIVTYEG--------DHNHSLS

PpWRKY8 KLADIPPDDYS**WRKY**GQKP-IKGSPHPRG--------------------------YYKCSS---I----RGCP-------------ARKHVERSME--------DPT--------------MLIVTYEG--------EHNHPQS

PpWRKY9 KLADIPPDDYS**WRKY**GQKP-IKGSPHPRG--------------------------YYKCSS---I----RGCP-------------ARKHVERSME--------DPT--------------MLIVTYEG--------EHNHPQL

PpWRKY2 KLADIPPDDYS**WRKY**GQKP-IKGSPHPRG--------------------------YYKCSS---I----RGCP-------------ARKHVERSME--------DPT--------------MLIVTYEGNHLHRTQTTHNHRLR

PpWRKY1 KLADIPSDEYS**WRKY**GQKP-IKGSPHPRG--------------------------YYKCSS---I----RGCP-------------ARKHVERSME--------DSS--------------MLIVTYEG--------DHNHPQS

AtWRKY21 KVADIPPDDYS**WRKY**GQKP-IKGSPYPRG--------------------------YYKCSS---M----RGCP-------------ARKHVERCLE--------DPA--------------MLIVTYEA--------EHNHPKL

OsWRKY25 RVADIPADEYS**WRKY**GQKP-IKGSPYPRG--------------------------YYRCST---V----KGCP-------------ARKHVERAAD--------DPA--------------TLVVTYEG--------DHRHSPP

OsWRKY44 RVADIPADEYS**WRKY**GQKP-IKGSPYPRG--------------------------YYRCST---V----KGCP-------------ARKHVERAAD--------DPA--------------TLVVTYEG--------DHRHSPP

OsWRKY51 KVADIPADDFS**WRKY**GQKP-IKGSPFPRG--------------------------YYKCST---L----RGCP-------------ARKHVERDPA--------DPS--------------MLIVTYEG--------EHRHTPS

HvWRKY10 KAAEIPADDFS**WRKY**GQKP-IKGSPYPRG--------------------------YYKCST---V----RGCP-------------ARKHVERDPS--------DPS--------------MLIVTYEG--------EHRHSPA

OsWRKY42 RNADIPADDYS**WRKY**GQKP-IKGSPYPRG--------------------------YYKCST---V----RGCP-------------ARKHVERDPG--------EPA--------------MLIVTYDG--------DHRHGEP

**Group2e**

OsWRKY37 SGEVVPSDLWA**WRKY**GQKP-IKGSPYPRG--------------------------YYRCSS---S----KGCS-------------ARKQVERSRT--------DPN--------------MLVITYTS--------EHNHPWP

OsWRKY66 SGEVVPSDLWA**WRKY**GQKP-IKGSPYPRG--------------------------YYRCSS---S----KGCS-------------ARKQVERSRT--------DPN--------------MLVITYTS--------EHNHPWP

AtWRKY35 SGEVVPSDLWA**WRKY**GQKP-IKGSPYPRG--------------------------YYRCSS---S----KGCS-------------ARKQVERSRT--------DPN--------------MLVITYTS--------EHNHPWP

AtWRKY14 SGEVVPSDLWA**WRKY**GQKP-IKGSPFPRG--------------------------YYRCSS---S----KGCS-------------ARKQVERSRT--------DPN--------------MLVITYTS--------EHNHPWP

OsWRKY2 GGEVVPSDLWA**WRKY**GQKP-IKGSPYPRG--------------------------YYRCSS---S----KGCS-------------ARKQVERSRA--------DPT--------------MLVVTYTS--------DHNHPWP

HvWRKY39 GEGPPPPDSWA**WRKY**GQKP-HQGSPYPRG--------------------------YYRCSS---F----KGCP-------------ARKQVERSRT--------DPT--------------VLLVTYSY--------DHNHPWP

OsWRKY13 GEGPPPSDSWA**WRKY**GQKP-IKGSPYPRG--------------------------YYRCSS---S----KGCP-------------ARKQVERSRA--------DPT--------------VLLVTYSF--------EHNHPWP

AtWRKY65 GDTTPPSDSWA**WRKY**GQKP-IKGSPYPRG--------------------------YYRCSS---T----KGCP-------------ARKQVERSRD--------DPT--------------MILITYTS--------EHNHPWP

AtWRKY69 GEVYPPSDSWA**WRKY**GQKP-IKGSPYPSGGLEMRKRRVALTVVG--------LTGYYRCSS---S----KGCP-------------ARKQVERSRV--------DPS--------------KLMITYAC--------DHNHPFP

OsWRKY14 GEGNTPTDSWA**WRKY**GQKP-IKGSPFPRA--------------------------YYRCSS---S----KGCP-------------ARKQVERSRN--------DPD--------------TVIVTYSF--------EHNHSAT

HvWRKY45 GEGNTPTDSWA**WRKY**GQKP-IKGSPFPRA--------------------------YYRCSS---S----KGCP-----------------------------------------------------------------------

OsWRKY12 ADGGVSSDLWA**WRKY**GQKP-IKGSPYPRG--------------------------YYKCSS---M----KGCM-------------ARKMVERSPA--------KPG--------------MLVVTYMA--------EHCHPVP

AtWRKY16 DRGSRSSDLWV**WRKY**GQKP-IKSSPYPRS--------------------------YYRCAS---S----KGCF-------------ARKQVERSRT--------DPN--------------VSVITYIS--------EHNHPFP

OsWRKY39 PADGVSADVWA**WRKY**GQKP-IKGSPYPRG--------------------------YYRCSS---S----KGCP-------------ARKQVERSRS--------DPN--------------TFILTYTG--------EHNHSAP

HvWRKY44 AADGTSADPWA**WRKY**GQKP-IKGSPYPRG--------------------------YYRCST---D----KACE-------------ARKMVERCRD--------DPN--------------SFILTYTGG-------EHSHPAP

AtWRKY22 AAEALNSDVWA**WRKY**GQKP-IKGSPYPRG--------------------------YYRCST---S----KGCL-------------ARKQVERNRS--------DPK--------------MFIVTYTA--------EHNHPAP

AtWRKY27 TQENLSSDLWA**WRKY**GQKP-IKGSPYPRN--------------------------YYRCSS---S----KGCL-------------ARKQVERSNL--------DPN--------------IFIVTYTG--------EHTHPRP

AtWRKY29 KEENLLSDAWA**WRKY**GQKP-IKGSPYPRS--------------------------YYRCSS---S----KGCL-------------ARKQVERNPQ--------NPE--------------KFTITYTN--------EHNHELP

OsWRKY31 AASGPAPDLWA**WRKY**GQKP-IKGSPYPRG--------------------------YYRCSS---N----KNCA-------------ARKQVERCRF--------DPS--------------FLLLTYTGA-------HSGHDVP

**GROUP3**

OsWRKY63_C VTSVPADDGYS**WRKY**GQKN-VLGFSYL--------------------------RGYYRCAT--------KGCQ-------------ASKQVQRHD---------DGL--------------LFDVTYFG--------EHTCADQ

HvWRKY24_C QEDYPADDGYS**WSKY**GQMD-ILGSKHP--------------------------RCYYRCVHKHD-----KGCQ-------------ATKQVQRSDS--------DTQ--------------LFDIVYHG--------EHTCAEN

OsWRKY61_N RMSYTEDDGFS**WRKY**GQKD-VEGAMHPTTQ----------------------SKSYFRCAHKMT-----TGCK-------------ARKKVQRTDG--------DPL--------------MVDVVYKG--------VHSCAGV

OsWRKY81_N RMSYTEDDGFS**WRKY**GQKD-VEGAMHPTTQ----------------------SN-YFRCAHKMT-----TGCK-------------ARKKVQRTDG--------DPL--------------MVDVVYKG--------VHSCAGV

OsWRKY41_N KVSSTVEDGFS**WVKY**GQKD-ILGTMYP--------------------------RSYFRCIHRHT-----KGCL-------------ATKQVQPTDD--------DHQ--------------ILDVIYYG--------EHTCDQS

OsWRKY63_N VRSNTEDDGLS**WSKY**EQKE-ILGAKFP--------------------------RAYFRCTHWNTK----KGCM-------------ATKEVQRDDG--------DPL--------------MFDIVYHG--------EHTCTQT

OsWRKY61_C KDVGPPDDGYS**WKRY**GQKN-IFGANYP--------------------------RCYYRCIHK-TT----TGCT-------------ATKNAQATDG--------DPL--------------LFDVVYHG--------EHTCDLQ

OsWRKY81_C KDVGPPDDGYS**WKRY**GQKN-IFGANYP--------------------------RCYYRCIHK-TT----TGCT-------------ATKNAQATDG--------DPL--------------LFDVVYHG--------EHTCDLQ

HvWRKY24_N QDIEGHDDGFS**WRKY**GQKD-ILGSRYP--------------------------RRYYRCKHR-LT----QGCE-------------AVKQLQATDG--------DPL--------------LFNAMYVG--------NHICIQR

HvWRKY25 QDIEGHDDGFS**WRKY**GQKDNIFGSKYP--------------------------RGYYKCNH---A----RGCP-------------ARKELQATDG--------DPL--------------LFDATYVG--------NHTCAHG

OsWRKY15 QDVGPLDDGFS**WRKY**GQKD-ILGAKYP--------------------------RAYFRCTHRHT-----QGCH-------------ASKQVQRADG--------DPL--------------LFDVVYHG--------DHTCAHG

HvWRKY21 EDVGPLDDGFS**WRKY**GQKD-ILGAMYP--------------------------RAYFRCTHRQA-----QGCY-------------ASKQVQRAHG--------DPL--------------LFDVVYHG--------NHTCAQG

HvWRKY26 HDLGPLDDGLS**WRKY**GQKD-ILGATYP--------------------------RAYFRCTHRHS-----QGCQ-------------ATKQVQRAHA--------DPL--------------LFDVVYHG--------AHTCAQ-

HvWRKY27 TDVGPLNDGLS**WRKY**GQKD-ILGATYP--------------------------RAYFRCTHRHT-----QGCQ-------------ATKQVQRTHA--------DP--------------------------------------

OsWRKY19 QDTASLDDGLS**WRKY**GQKD-ILGAKYP--------------------------RAYFRCTHRHT-----QGCN-------------ATKQVQRADG--------DPL--------------LFDVVYLG--------DHTCGQA

OsWRKY41_C QDVGPLDDGYS**WRRY**GLKD-ILGAKYP--------------------------RSYFRCTHRNT-----QGCV-------------ATKQIQRRDG--------DPL--------------LFDVVYHG--------DHTCSER

HvWRKY29 QDLGPLDDGMS**WRKY**GQXG-ILGATYP--------------------------RSYFRCTHR----------------------------------------------------------------------------------

OsWRKY69 GAEGPADDGHS**WRKY**GQKD-ILGAKHP--------------------------RGYYRCTHRNT-----QGCT-------------ATKQVQRTDD--------DAS--------------LFDVVYHG--------EHTCRPG

HvWRKY30 GAEGPGDDGHS**WRKY**GQKD-ILGAKHP--------------------------RAYYRCTHRNS-----QGCT-------------ATKQVQRADE--------DPV--------------LFDVVYHG--------QHTCRPT

HvWRKY22 GAETPVDDGHS**WRKY**GQKD-ILGAKHP--------------------------RGYYRCTHRXS-----QGCA-------------ATKQVQRADE--------DPT--------------LFDVIYHG--------EHTCIH-

HvWRKY28 GADAPADDGHS**WRKY**GQKD-ILGAHHP--------------------------RAYYRCTYQKT-----QGCA-------------ATKQVQRADE--------DPA--------------LFDVIYHG--------EHTCLHK

OsWRKY74 VENPPVDDGHS**WRKY**GQKE-ILGAKHP--------------------------RGYYRCTHRHS-----QGCM-------------ATKQVQRTDE--------DAM--------------VFDVIYHG--------EHTCVHK

AtWRKY41 GLEGPHDDIFS**WRKY**GQKD-ILGAKFP--------------------------RSYYRCTFRNT-----QYCW-------------ATKQVQRSDG--------DPT--------------IFEVTYRG--------THTCSQG

AtWRKY53 GLEGPQDDVFS**WRKY**GQKD-ILGAKFP--------------------------RSYYRCTHRST-----QNCW-------------ATKQVQRSDG--------DAT--------------VFEVTYRG--------THTCSQA

AtWRKY30 GVDRTLDDGFS**WRKY**GQKD-ILGAKFP--------------------------RGYYRCTYRKS-----QGCE-------------ATKQVQRSDE--------NQM--------------LLEISYRG--------IHSCSQA

OsWRKY48 EKARTSEDGFL**WRKY**GQKE-IKNSKH--------------------------PRLYYRCSYKDD-----HGCT-------------ATKQVQQSE--------EDPS--------------LYVITYFG--------DHTCSCQ

OsWRKY54 ERKATMDDKFL**WRKY**GQKE-IKNSKH--------------------------PRFYYRCSYKDD-----HGCT-------------ATKQVQQSETA-----DDDTASP------------VYIITYFG--------EHTCRHG

HvWRKY31 ERRRTAEDGLI**WRKY**GQKE-IHNSTH--------------------------PRLYFRCTYKHD-----SGCP-------------ATRQVQQSE--------DDPS--------------LYVITY-----------------

OsWRKY21 VRSGTTTDGFI**WRKY**GQKE-INGCKH--------------------------PRLYYRCAFR-G-----QGCL-------------ATRRVQQSQS------QDDPAA-------------AFVIAYYG--------EHTCGGD

OsWRKY47 VTTKELEDGRQ**WRKY**GQKH-IQDSPNN-------------------------PRSYYRCTHRPD-----QGCM-------------ATKQVQTSE--------SNSS--------------EFVISYYG--------EHTCRDP

HvWRKY4 VTARTLNDGKT**WRKY**GQKC-IHACTN--------------------------PRSYYRCSHKPD-----QGCQ-------------ATRQVQESD--------SSPS--------------EYLISYYG--------QHTCKDP

OsWRKY45 VVVKNLDDGQA**WRKY**GQKE-IQNSKH--------------------------PKAYFRCTHKYD-----QMCT-------------AQRQVQRCD--------DDPA--------------SYRVTYIG--------EHTCRDP

HvWRKY32 KKMKSLEDGQT**WRKY**GQKE-IQNSKH--------------------------SKAYFRCTHKYD-----QQCP-------------ARRQAQRCD--------EDPD--------------TYRVTYIG--------VHTCQDP

AtWRKY54 VEAKSSEDRYA**WRKY**GQKE-ILNTTF--------------------------PRSYFRCTHKPT-----QGCK-------------ATKQVQKQD--------QDSE--------------MFQITYIG--------YHTCTAN

AtWRKY70 IESTILEDAFS**WRKY**GQKE-ILNAKF--------------------------PRSYFRCTHKYT-----QGCK-------------ATKQVQKVE--------LEPK--------------MFSITYIG--------NHTCNTN

AtWRKY46 QENGSIDDGHC**WRKY**GQKE-IHGSKN--------------------------PRAYYRCTHRFT-----QDCL-------------AVKQVQKSD--------TDPS--------------LFEVKYLG--------NHTCNNI

OsWRKY75 NTEQPPDDGYT**WRKY**GQKD-ILGSRY--------------------------PRSYYRCTHKNY-----YGCE-------------AKKKVQRLDD--------DPF--------------TYEVTYCG--------NHTCLTS

AtWRKY55 NTDLPPDDNHT**WRKY**GQKE-ILGSRFPR--------------------------AYYRCTHQKL-----YNCP-------------AKKQVQRLND--------DPF--------------TFRVTYRG--------SHTCYNS

OsWRKY40 ETPVPHYDGHQ**WRKY**GQKH-IKNSKHPR--------------------------SYYRCTYRQE-----EKCK-------------ATKTVQQREDLHHANSYNGDHPI------------MYTVVYYG--------QHTCCKG

OsWRKY64 ETPVPHYDGHQ**WRKY**GQKH-INNSKHPR--------------------------SYYRCTYRQE-----EKCK-------------ATKTVQQREDLHHANSYNGDHPV------------MYTVVYYG--------QHTCCKG

OsWRKY18 LTPIPHTDGHL**WRKY**GEKK-IKNSSFPR--------------------------LYYRCSYRDD-----RNCM-------------ATKVVQQE---------NDADPP------------LYRVTYIH--------PHTCNPS

OsWRKY46 DTYAPYDDGHQ**WRKY**GEKK-LSNSNFP------------------------------RCTYKND-----MKCP-------------ATKQVQQK---------DTNDPP------------LFSVTYFN--------HHTCNSS

HvWRKY34 DTYAPYDDGHQ**WRKY**GEKK-LSNSNFPR--------------------------FYYRCTYKTD-----LKCP-------------ATKQVQQK---------DMSDPP------------LFTVTYFN--------HHSCNTT

HvWRKY33 DTFAPHNDGHQ**WRKY**GEKK-INNCNFPR--------------------------YYYRCTYKDN-----MNCP-------------ATKQIQQK---------DHSDPP------------LYQVTYYN--------EHSCNSA

OsWRKY55 DTFAPHNDGHQ**WRKY**GEKK-INNCNFP----------------------------------RTN-----DQC---------------------------------------------------QLTFFK--------RSTA---

OsWRKY52 FTPVPHEDGFQ**WRKY**GEKK-IQGTHFT------------------------------SVNTQED-----DGGV-------------AMDS------------------------------------------------------

OsWRKY22 YTYAPYHDGYQ**WRKY**GQKM-IRGNSFPR--------------------------CYYRCTYHQD-----HGCP-------------ASKHVEQH---------NSEDPP------------LFRVIYTN--------EHTCGTS

OsWRKY20 ITPSPYKDGYQ**WRKY**GQKN-IQDSNYLR--------------------------LYFKCTFSRE-----RSCA-------------AKKQVQQR---------DAGEPP------------MFLVTYLN--------EHTCQQP

OsWRKY50 VTTVPDFDGYQ**WRKY**GQKQ-IEGAMYPR--------------------------SYYRCTNSTN-----QGCL-------------AKKTVQRN---------GGGGAA------------GYTVAYIS--------EHTCKSI

OsWRKY65 VTTVPDFDGYQ**WRKY**GQKQ-IEGAMYPR--------------------------SYYRCTNSTN-----QGCL-------------AQKTVQRN---------GGGGAA------------GYTVAYIS--------EHTCKSI

AtWRKY38 SPDPIYYDGYL**WRKY**GQKS-IKKSNHQR--------------------------SYYRCSYNKD-----HNCE-------------ARKHEQKIKD--------NPP--------------VYRTTYFG--------HHTCKTE

AtWRKY62 SSTPIYHDGFL**WRKY**GQKQ-IKESEYQR--------------------------SYYKCAYTKD-----QNCE-------------AKKQVQKIQH--------NPP--------------LYSTTYFG--------QHICQLH

AtWRKY63 SPNPRLDDGFT**WRKY**GQKT-IKTSLYQR--------------------------CYYRCAYAKD-----QNCY-------------ATKRVQMIQD--------SPP--------------VYRTTYLG--------QHTCKAF

AtWRKY64 SPTPRPDDGFT**WRKY**GQKT-IKTSPYQR--------------------------CYYRCTYAKD-----QNCN-------------ARKRVQMIQD--------NPP--------------VYRTTYLG--------KHVCKAV

AtWRKY67 SRTMCPNDGFT**WRKY**GQKT-IKASAHKR--------------------------CYYRCTYAKD-----QNCN-------------ATKRVQKIKD--------NPP--------------VYRTTYLG--------KHVCKAF

AtWRKY66 SPTPAHIDGFI**WRKY**GQKT-IKTSPHQR--------------------------WYYRCAYAKD-----QNCD-------------ATKRVQKIQD--------NPP--------------VYRNTYVG--------QHACEAP

AtWRKY52 IPAIDEGDLWT**WRKY**GQKD-ILGSRFPR--------------------------GYYRCAYK-FT----HGCK-------------ATKQVQRSET--------DSN--------------MLAITYLS--------EHNHPRP

**Outgroups**

DdWRKY_N NISNIVSDGYQ**WRKY**GQKN-VKGSLHP--------------------------RHYYKCTF--------QGCN-------------VRKQVERIGD--------TNQ---------------NSTVYKG--------EHCHGFP

DdWRKY_C SSIDHLDDGFF**WRKY**GQKS-VKGSPF---------------------------KSYFKCAELT--------CP-------------VKKQVIQQDSK-------------------------YINTYRG--------KHNHDPP

GlWRKY_N TIRELPADGYC**WRKY**GSKRLPNNSHP---------------------------KSYFRCSV--------PGCQ-------------AKRYVTETD---------NRV---------------LKTEYIG--------EHNHGKS

GlWRKY_C DQIESSIDFFR**WKKY**GHKPQTDTRLDS--------------------------KSYYRCAFFN--------CP-------------ARRTITFFYSL-------SSDGTETVES--------VIVQYEN--------QHTHPPD

HsFLYWCH GGRLLVLESFLYKQEKAVGD---------------------------------KVYWKCRQHAE-----LGCR-------------GRAITRG-----------------------------LRATVMR--------GHCHAPD

MmFLYWCH GGRLLVHKSFLYKQEKAVGD---------------------------------KVYWKCRQHSE-----LSCR-------------GRAITRG-----------------------------FRVTEMR--------DHCHPPE

At2g02190 LRMFAVKHSFEFHTVKSDLT---------------------------------RYVLHCID--------ENCS-------------WRLRATRAGG--------SES---------------YVIRKYV-------SHHNCDSS
