# Supplementary material for: Phylogenetic and comparative gene expression analysis of barley (Hordeum vulgare) WRKY transcription factor family reveals putatively retained functions between monocots and dicots
Source: BMC Genomics. 2008 Apr 28;9:194. doi: 10.1186/1471-2164-9-194 (PMC2390551; doi:10.1186/1471-2164-9-194)

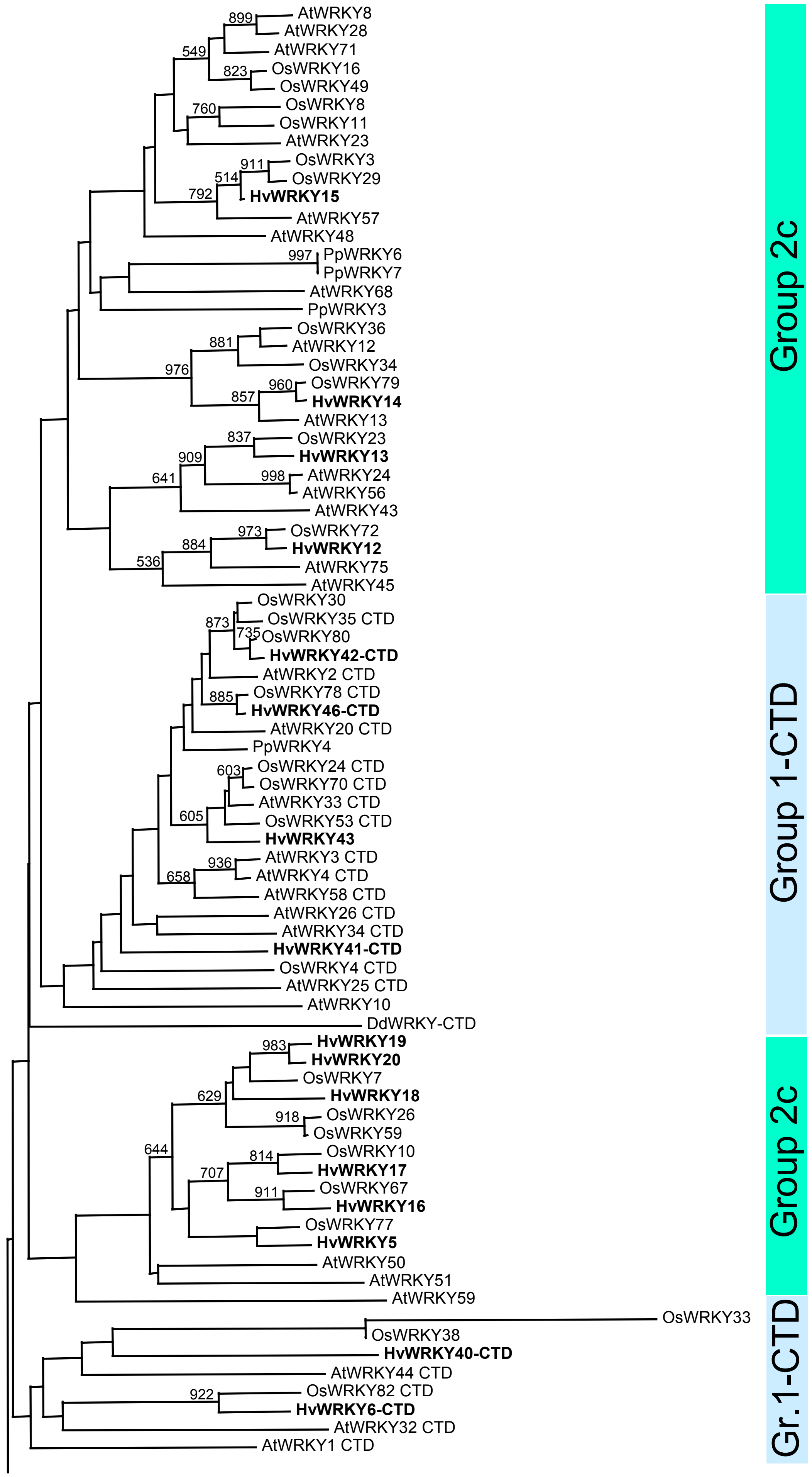

continued on page 2

continued on page 1

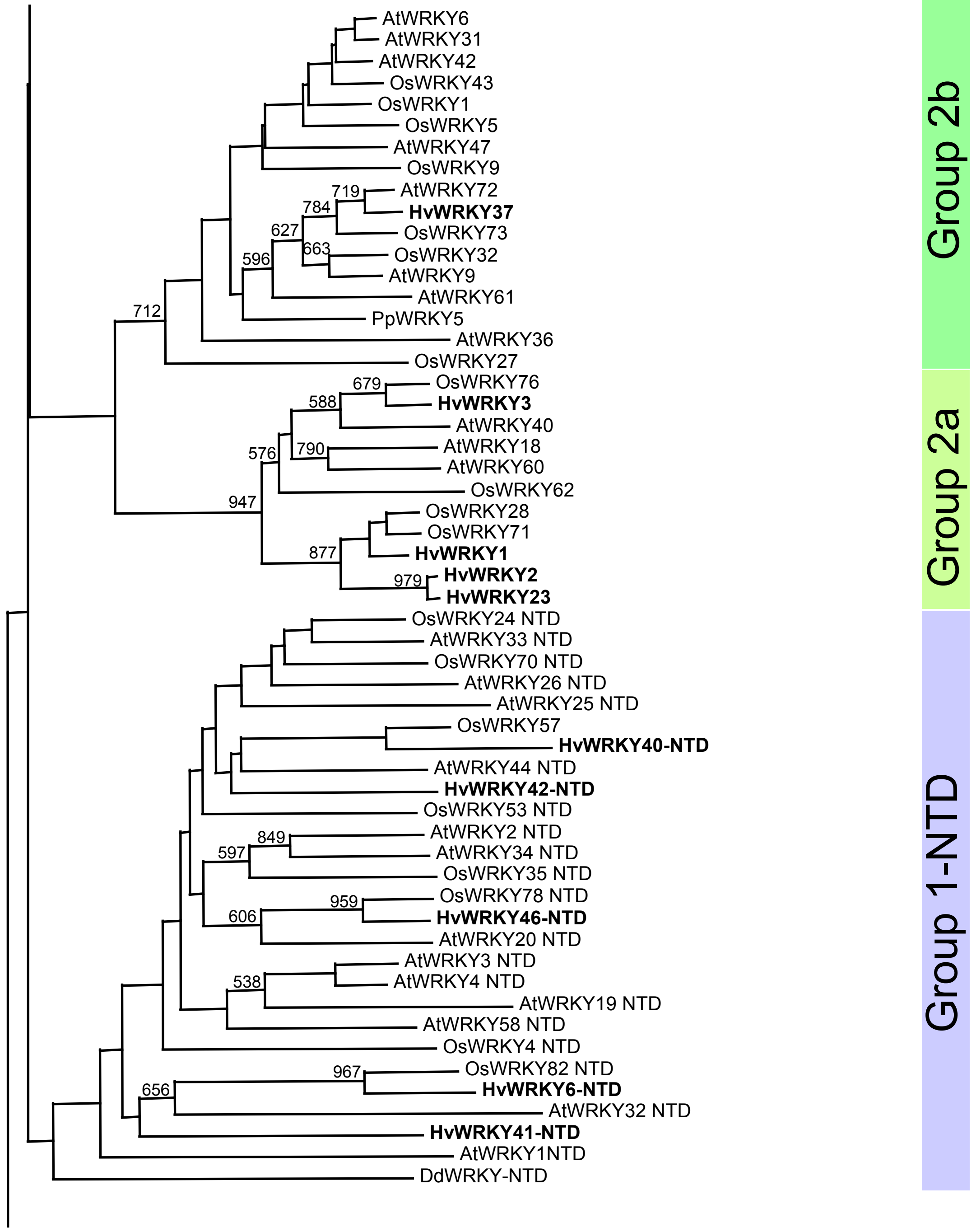

continued on page 3

continued on page 2

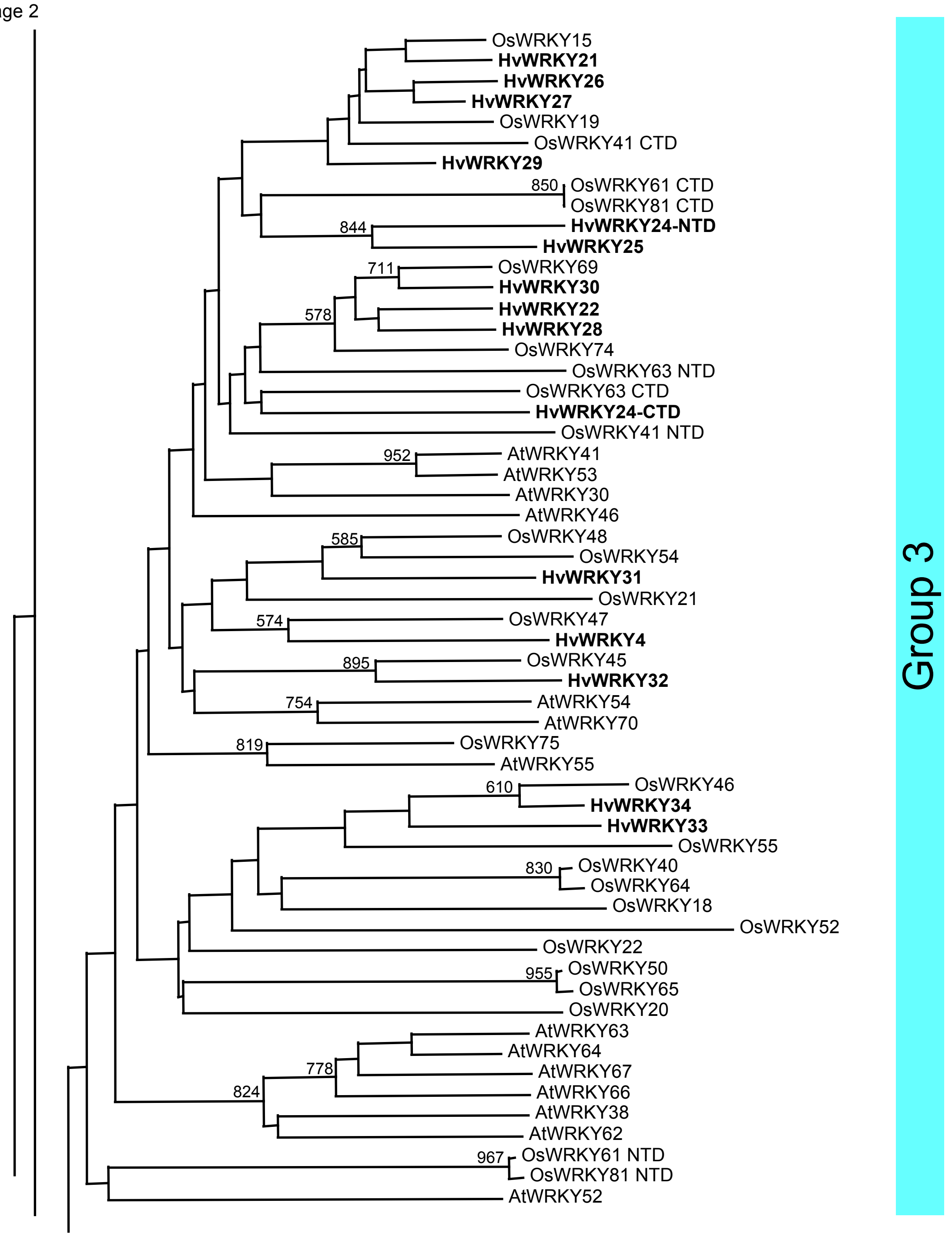

continued on page 4

continued on page 3

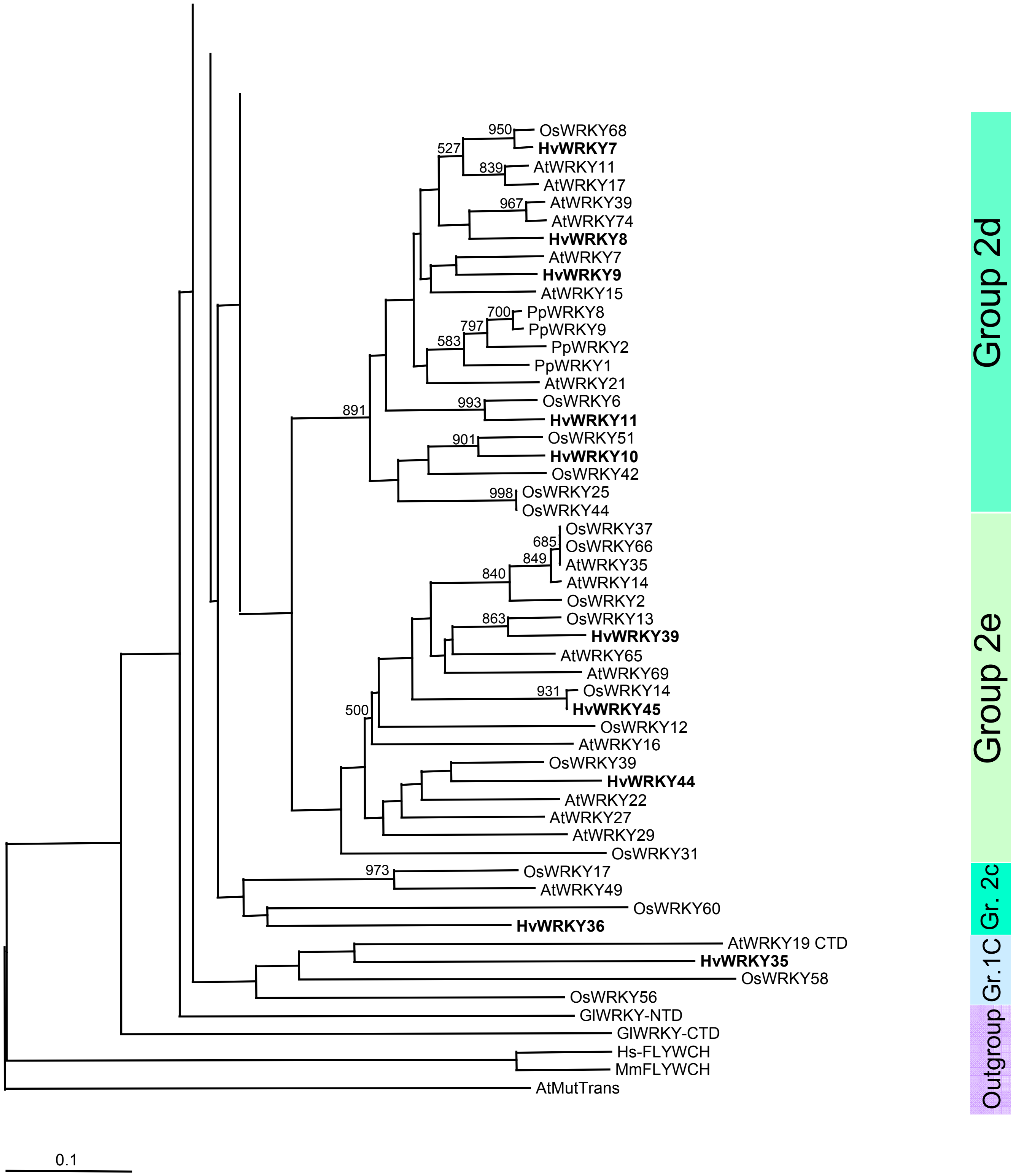

Supplement: Additional File 2 — Phylogenetic tree based on all WRKY_GCM1-like domains considered in the Alignment of Additional File 1. Phylogenetic tree based on the WRKY domain amino acid sequences given in Additional Figure 1. Sequences of Arabidopsis (AtWRKY), barley (HvWRKY), Physcomitrella (PpWRKY), rice (OsWRKY), Dictyostelium discoideum (DdWRKY), Giardia lamblia (GlWRKY) Homo sapiens (HsFLYWCH) and Mus musculus (MmFLYWCH) are arranged in clusters with the zinc finger domain of the Arabidopsis mutator-like transposase (AtMutTrans) as outgroup. Tree topology has been confirmed by using three different programs. Bootstrap values from 1000 iterations and bigger than 500 are included in the tree. HvWRKY sequences are highlighted in bold letters. Groups and subgoups of WRKY1 to 3 are indicated by bars on the right side. [file 1471-2164-9-194-S2.pdf]
