# Supplementary figures and images for: Phylogenetic and comparative gene expression analysis of barley (Hordeum vulgare) WRKY transcription factor family reveals putatively retained functions between monocots and dicots
Source: BMC Genomics. 2008 Apr 28;9:194. doi: 10.1186/1471-2164-9-194 (PMC2390551; doi:10.1186/1471-2164-9-194)

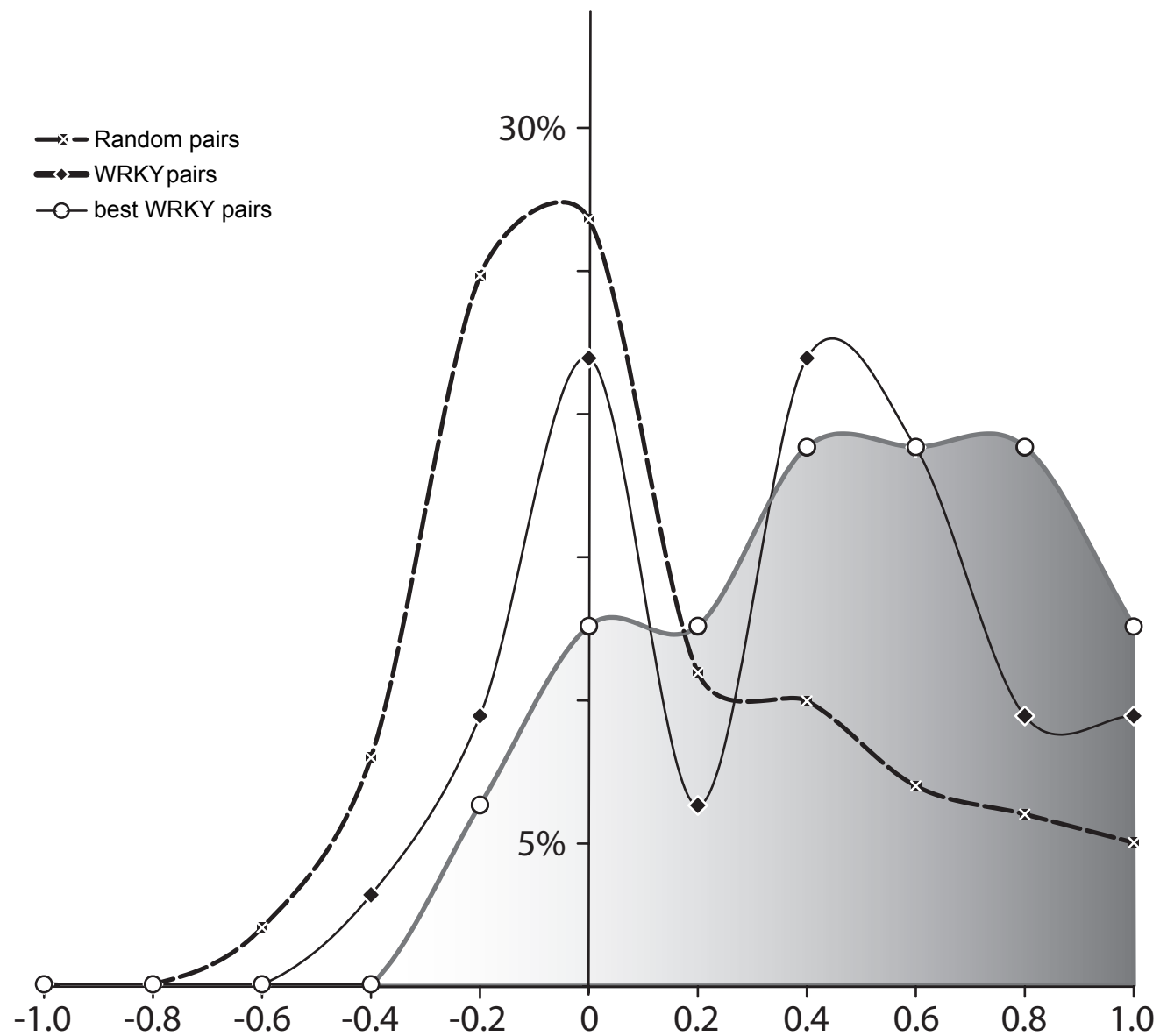

Supplement: Additional File 5 — Distribution of the relative correlation coefficient in randomly chosen and HvWRKY-AtWRKY gene pairs. The relative correlation coefficient was calculated from the normalized signal intensities of the gene pairs and plotted according to its occurrence. Random gene pairs exhibited an average correlation coefficient of 0.01. WRKY gene pairs exhibited a divided curve with two maxima. When only the best pairing orthologs were considered, the first peak of more random distribution is lost. [file 1471-2164-9-194-S5.pdf]
